# Automated identification of reference genes based on RNA-seq data

**DOI:** 10.1186/s12938-017-0356-5

**Published:** 2017-08-18

**Authors:** Rosario Carmona, Macarena Arroyo, María José Jiménez-Quesada, Pedro Seoane, Adoración Zafra, Rafael Larrosa, Juan de Dios Alché, M. Gonzalo Claros

**Affiliations:** 1 Plant Reproductive Biology Laboratory, Department of Biochemistry, Cell and Molecular Biology of Plants, Estación Experimental del Zaidín, CSIC, Granada, Spain; 2 Servicio de Neumología, Hospital Regional Universitario de Málaga, Avda Carlos Haya s/n, Malaga, Spain; 3 Departamento de Biología Molecular y Bioquímica, Universidad de Málaga, Malaga, Spain; 4 Departamento de Arquitectura de Computadores, Universidad de Málaga, Malaga, Spain

**Keywords:** Reference genes, Normalization, Real-time PCR, Quantitative PCR, Olive (*Olea europaea* L.), Cancer

## Abstract

**Background:**

Gene expression analyses demand appropriate reference genes (RGs) for normalization, in order to obtain reliable assessments. Ideally, RG expression levels should remain constant in all cells, tissues or experimental conditions under study. Housekeeping genes traditionally fulfilled this requirement, but they have been reported to be less invariant than expected; therefore, RGs should be tested and validated for every particular situation. Microarray data have been used to propose new RGs, but only a limited set of model species and conditions are available; on the contrary, RNA-seq experiments are more and more frequent and constitute a new source of candidate RGs.

**Results:**

An automated workflow based on mapped NGS reads has been constructed to obtain highly and invariantly expressed RGs based on a normalized expression in reads per mapped million and the coefficient of variation. This workflow has been tested with Roche/454 reads from reproductive tissues of olive tree (*Olea europaea* L.), as well as with Illumina paired-end reads from two different accessions of *Arabidopsis thaliana* and three different human cancers (prostate, small-cell cancer lung and lung adenocarcinoma). Candidate RGs have been proposed for each species and many of them have been previously reported as RGs in literature. Experimental validation of significant RGs in olive tree is provided to support the algorithm.

**Conclusion:**

Regardless sequencing technology, number of replicates, and library sizes, when RNA-seq experiments are designed and performed, the same datasets can be analyzed with our workflow to extract suitable RGs for subsequent PCR validation. Moreover, different subset of experimental conditions can provide different suitable RGs.

**Electronic supplementary material:**

The online version of this article (doi:10.1186/s12938-017-0356-5) contains supplementary material, which is available to authorized users.

## Background

Traditionally, gene expression studies have been carried out by non-quantitative or semi-quantitative RNA gel blotting and later by reverse transcription-polymerase chain reaction (RT-PCR) analyses. Development of real-time, quantitative PCR (qPCR) [1] took the place of these techniques due to its higher specificity, sensitivity and broad quantification range. The use of an appropriate reference gene (RG) to avoid false results and for proper interpretation of gene expression data soon emerged as a significant concern in these experiments, mainly due to the increased sensitivity of qPCR with respect to Northern blotting and RT-PCR. The first RGs were brought from Northerns and usually encoded proteins involved in structural functions and basic cell metabolism due to their theoretical expression invariability in most tissues. This initial election was revealed inappropriate [2–4] and the quest of more reliable RGs has been pursued in the literature [5–8].

Conclusions of any qPCR experiment are depending on RGs, but also on the selection of an appropriate normalization method. Relative quantification is the most widely used method for normalization, where gene expression level is normalized by an internal RG that should remain constant in all experimental conditions under study. BestKeeper [9], geNorm [10] and NormFinder [11] are the most popular methods for normalization and confirming RGs. Based on the raw, relative quantities, geNorm calculates the minimal number of RGs for each experiment and NormFinder also provides a stability value for each gene. BestKeeper employs a pair-wise correlation analysis based on a geometric mean to determine the optimal RGs. But all of them are based on qPCR data, which produce a recursive problem since a qPCR is required to decide if a RG is appropriate for qPCR. Reports describing that several of the most commonly used housekeeping genes exhibit substantial variability in microarray data sets or under different experimental conditions are becoming more and more frequent [12, 13]. Consequently, the choice of the best RGs should be based on preliminary experimental evidence when comparing different developmental stages, tissues, cell types or environmental conditions, as well as in careful testing and validation [14]. Hence, the selection of both RGs and normalization method are critical for obtaining reliable quantitative gene expression assessments to correct for non-specific variation, such as differences in RNA quantity and quality.

In the search for appropriate, stable RGs, a data mining strategy based on the use of publicly available microarray data repositories was envisaged. It was available only for some species, usually model organisms [15], and provided useful RGs [7]. When microarray data are unavailable (i.e. for non-model organisms or unusual experimental conditions), other strategies must be regarded to establish suitable RGs. Such is the case of the olive tree (*Olea europaea* L.), one of the most important oil-producing plant species all over the world. Although a first draft genome of this plant has just been published [16] and some gene expression analyses have been reported [17, 18], further and longer studies will be required to select reliable RGs. Several attempts for the identification of putative RGs in this species have been carried out, by evaluating olive genes orthologs to the best-ranked RGs from other crops. They were selected according to their stability in olive tissues, as it occurs in other plants, and throughout different experimental conditions: different developmental stages of the olive mesocarp tissue across different cultivars [19], and several fruit developmental/ripening stages and leaves subjected to wounding [20, 21]. The peculiarity of plant reproductive tissues makes the search of these RGs particularly tricky, as some well known housekeeping genes display differential expression in pistil, pollen and other floral organs [15]. Nevertheless, other analyses indicate that a large proportion of constitutive transcripts are shared by most somatic, reproductive, and haploid tissues [22]. Consequently, a reasonable thought is that RGs can be more easily detected in model organism such as *Arabidopsis*, mice or humans, where more microarray data are available. The only problem with these species relies on the experimental conditions for which new RGs are required. It can be concluded that ideally RGs for qPCR validation could be inferred from the experimental data to be analyzed.

The falling cost of NGS (next-generation sequencing) technologies has made their use more and more frequent. This has resulted in an explosive growth of data that are gathered into the Sequence Read Archive (SRA) [23]. This public repository allows for new discoveries by comparing the archived data sets. Since any RNA-seq study requires further, experimental validation, and qPCR has become the de facto standard, we thought that NGS data can also be analyzed as a source of RGs. With this aim, an automatic workflow has been constructed to obtain highly, but invariantly, expressed RGs for particular experimental conditions based on the coefficient of variation (CV) of normalized expression values by RPMM (reads per mapped million) and managing both Roche/454 and Illumina reads. Candidate RGs have been proposed for reproductive tissues of olive tree, *Arabidopsis thalian*a flowers, and three different human cancers. Experimental validation of olive tree RGs is also included.

## Methods

### Sequence reads and reference transcriptomes

*Olea europaea* (olive tree) reads (SRA BioProject PRJNA287107) correspond to a Roche GS-FLX Titanium + sequencing experiment for different developmental stages of pollen and pistil, as described in [17]. Reads (mean length 385 nt) were mapped only against transcripts coding for a complete protein (9157 transcripts) in the reproductive transcriptome described in ReprOlive (http://reprolive.eez.csic.es) [17].

*Arabidopsis thaliana* reads were obtained from SRA BioProject PRJEB9470. A late flowering strain (Columbia) and the reference Killean were compared to determine genes involved in early flowering [24]. Three biological replicates from ten day-old seedlings were paired-end sequenced (100 bp) on a HiSeq 1000. The *Arabidopsis* reference transcriptome (35,386 transcripts) was downloaded from Phytozome (https://phytozome.jgi.doe.gov) and refers to TAIR10 [25].

Sequencing reads from matched normal and malignant tissues from the same patient were considered for the study in humans. Matched normal and malignant prostate tissues from 14 Chinese [26] were obtained from SRA BioProject PRJEB2449 (HiSeq 2000, 90 nt paired-end reads). Sequencing of matched samples of normal lung and small-cell lung cancer of 17 patients [27] were available under permission at EGA under accession EGAS00001000334 (HiSeq 2000, 75 nt, paired-end reads). Matched samples of normal lung and lung adenocarcinoma from 50 patients [28] were downloaded from ENA under accession number ERP001058 (HiSeq 2000, 100 nt, paired-end reads). The three data sets of human reads were mapped onto the cDNA data set of 176,241 transcripts (downloaded from the ENSEMBL repository) deduced from the human GRCh38 genome.

### Read count table

**Fig. 1:**
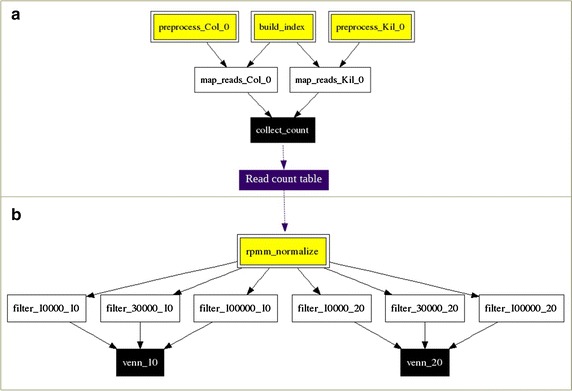
Flow diagram as provided by AutoFlow for the detection of RGs using the SRA datasets of PRJEB9470 from *Arabidopsis*. **a** The first workflow that prepares the reads, maps them on the transcriptome and provides the read count table. **b** The *findRGs* workflow for detecting candidate RGs. In this example, several filtering parameters were tested: 10 and 20% for the maximum CV, and 10,000, 30,000 and 100,000 reads for the minimum counted reads per transcript and condition. One *Venn diagram* by each CV cut-off is obtained, as shown in Figs. 2, 4, 5 and 6

A scheme of the automatic workflow executed in this work for obtaining the matrix of read counts of transcripts vs. experimental conditions is shown in Fig. 1a for the *Arabidopsis* datasets. It has been constructed using AutoFlow [29], a workflow manager developed in Ruby in our laboratory. The workflow receives as input files the raw reads and the transcriptome, both in Fasta format. Each file of raw reads is considered an experimental condition. Raw reads are then pre-processed using SeqTrimNext [30] to remove noisy sequences and retain only reliable reads. Useful reads are then mapped to the corresponding transcriptome using Bowtie2 [31] with default parameters and the −a option to allow each read to map in all possible transcripts. Mapped reads are then counted with Bio-samtools from BioRuby [32] with the −f2 option to count only reads where both ends are mapping on the same transcript (only for Illumina reads). The output is the tab-delimited, read count table where columns are experimental conditions, rows are transcripts, and the values are the number of counts of each transcript at each condition. Other pre-processing and mapping approaches can also be used provided that a tab-delimited read count table of transcripts vs. experimental conditions is obtained.

### Detection of reference genes

A second workflow (called *findRGs*, Fig. 1b) will look for the candidate RGs and is the main contribution of this work. It is also based on AutoFlow, although the basic functions could be also implemented in a spreadsheet. The input required are the previous read count table and, optionally, a tab-delimited annotation table with at least two columns: the first column containing the ID of each transcript, and the second column containing a reference ID of an orthologous gene. For convenience, it is recommended to add a third column containing the description of the ortholog. As shown in Fig. 1b, counts in the table are then normalized as the number of reads mapped on a transcript divided by the number of transcriptome-mapped reads of the corresponding sample (RPMM: reads per mapped million). Coefficient of variation (CV, ratio of the standard deviation to the mean, expressed as a percentage) of RPMM along all conditions is obtained per transcript. This normalized table containing the RPMMs and the CVs is analyzed by two customizable parameters: (1) CV (10% maximum, although the range can move from 0 up to 20) to select for genes whose expression is as invariant as possible, and (2) counted reads per transcript and condition (minimum of 10; we propose to start with a minimum value resulting from the multiplication of 0.00003 by the lowest library size) to select for RG with the highest level of expression to warrant the correct amplification by PCR. The workflow allows combining different values of these two parameters in a single execution. When the reference transcriptome used for mapping overestimates the number of transcripts, many of them will refer to the same gene. In this case, the optional annotation table serves to detect transcripts sharing the same ortholog or ID, and filter them to retain only the one with the highest RPMM value. Finally, a Venn diagram showing the number of specific and common orthologs between the different combinations of tested parameters is generated, in order to visualize the results and check the suitability of such parameters.

To install *findRGs*, first install Ruby 1.9.3 or higher and R 3.0.2 or higher, and then install AutoFlow, the workflow manager, as a gem with the command *gem install autoflow*. Other dependiencies have to be downloaded with the command *git clone* ‘https://github.com/seoanezonjic/general_scripts.git’, and then placed in a ‘custom’ directory. This custom directory containing AutoFlow scripts must be included in the $PATH environment variable of your computer as *export PATH* = *"path_to_custom_directory:$PATH"* in the.baschrc file. Finally download *findRGs* to the AutoFlow environment with the commad *AutoFlow* –*get_template_repository* ‘https://github.com/rosariocarmona/autoflow_templates.git’. The workflow can be executed as *AutoFlow* −*w findRGs* −*V ‘$input_file* = *read_count_table,$min_reads* = *[10;50;100],$cv_filter* = *[10, 20],$annot* = *annotation_table*, where −*w* indicates that *findRGs* is the workflow template to be executed and −*V* sets the default parameters.

### Experimental validation of candidates to RG in olive tree

Preliminary validation of some olive tree candidates was performed by semi-quantitative, real time PCR analysis according to Alché et al. [33] using 25 cycles to ensure an exponential amplification rate. Primers used are listed in Table 1. A total of 5 μl of the PCR reaction were loaded per lane and separated on 2% agarose gels in Tris–borate-EDTA (TBE). Equal loading of the RT mixture used for PCR was ensured by using Bioanalyzer (Agilent Technologies) accurate quantitation.

**Table 1:** Primers used for PCR amplification

Gene	Direction	Sequence
18S	Forward	5′-TTT GAT GGT ACC TGC TAC TCG GAT AAC C
	Reverse	5′-CTC TCC GGA ATC GAA CCC TAA TTC TCC
Ubiquitin monomer to pentamer	Forward	5′-ATGCAGAT(C/T)TTTGTGAAGAC
	Reverse	5′-ACCACCACG(G/A)AGACGGAG
Actin	Forward	5′-TTG CTC TCG ACT ATG AAC AGG
	Reverse	5′-CTC TCG GCC CCA ATA GTA ATA
Mitogen-activated protein kinase	Forward	5′-CCAGGCGAGATTTCAGAGAC
	Reverse	5′-TCGGTTTAAGGTCTCGATGG
Proline transporter	Forward	5′-TTGTAGTGAGGGGCGGTTAC
	Reverse	5′-CATGCAACCAAAGAAGCAGA
l-Ascorbate oxidase homolog	Forward	5′-ACAAAAGGCATTGCTTGGTC
	Reverse	5′-GGCCAAAACGAAGTTTACCA
Gliceraldehyde-3-phosphate dehydrogenase	Forward	5′-GGGCAAGATCAAGATTGGAA
	Reverse	5′-GTCTTCTCGCCGAACAAAAG
Salicylic acid-binding protein	Forward	5′-GCATTGACCCGAAAATCCTA
	Reverse	5′-AGGATGGCGGATTTGTAGTG
*S*-adenosylmethionine decarboxylase proenzyme	Forward	5′-AGCTTCTGGCATCAGGAAAA
	Reverse	5′-AGCCAGTACCCTCTCAAGCA

## Results and discussion

### Pre-processed reads

NGS reads cannot be used for mapping as obtained from the corresponding sequencing platform [30]. Therefore, they were pre-processed with SeqTrimNext and then mapped with Bowtie2. Dataset sizes collected for this study were intentionally very heterogeneous (Additional file 1) to test the workflow in different settings. The percent of useful reads seems to be homogeneous within Illumina reads (>64%, mean 85%, depending on datasets), as well as in 454/Roche reads (>50%, mean 54%). The percent of mapped reads with respect to useful reads is also homogeneous within technologies (last column on Additional file 1), suggesting the appropriateness of the pre-processing. Dataset size heterogeneity is regular within three samples: in olive tree, raw reads range from 217,163 to 262,749 (1.2 times) and useful reads from 111,760 to 150,185 (1.3 times); in *Arabidopsis*, raw reads range from 9,107,610 to 13,076,233 (1.4 times) and useful reads from 8,859,088 to 12,678,437 (1.4 times); and in prostate cancer, data are also very homogeneous, with a range of 1.2 times for both raw and useful reads (Additional file 1). But more extreme situations can be found on lung cancer samples: in small-cell lung cancer, raw reads range from 68.5 to 19.3 million raw reads (3.5 times) and useful reads from 16.8 to 62.6 million reads (3.72 times); and on adenocarcinoma, raw reads range from 10.9 to 109.3 million reads (5.5 times) and useful reads from 8.7 to 93.4 million reads (10.7 times). With such an heterogeneity, and in contrast to previously published [34], the transcript RNA abundance must be normalized within samples in order to remove the bias due to the sequencing depth of a sample.

Since no comparison between transcripts is performed, normalization by the length or the RNA species is not required. A widely used method of count normalization is RPKM (reads per kilobase per million reads) for single-end reads and the FPKM (single fragment per kilobase and million reads) [35], even though they have been revealed to be inconsistent for comparisons within the same sample [36]. However, in this work, gene expression is compared for one gene along all samples, making unnecessary that normalization by length, as each transcript count along samples will be divided by the same constant (transcript length). Moreover, in some non-model organisms (such as olive tree), the transcript length is not well known since only a fragment of the transcript has been reconstructed, or the transcript is divided in several independent contigs. That is why we have introduced a simplification of RPKM as the RPMM (reads per mapped million) based on the counts per million.

### Workflow execution times

Execution times for the different groups of tasks of the complete workflow (pre-processing, mapping and analysis with *findRGs*; Fig. 1) using three datasets from different species, different sequencing technology and increasing number of reads was assessed (Table 2). Using the same number of CPUs, the pre-processing task is by far the longest stage in olive tree and *Arabidopsis* (98% of total workflow time in olive tree and almost 70% in *Arabidopsis*), being much more lasting in olive tree pistil than in *Arabidopsis*, mainly due to the longer Roche/454 read length. In contrast, mapping is the longest task using human prostate reads (around 85% of total workflow time), due to the larger size of the transcriptome on which reads try to align. Analysis with *findRGs* is quite fast (below 1 min per 100,000 reads) in the three cases, regardless of read type or length, the species and the number of experimental conditions. Therefore, the analysing workflow is considerably fast and can be offered as a web tool, even though long reads or large transcriptomes might decrease its performance.

**Table 2:** Workflow execution times estimated for three datasets

Species/tissue	No. raw reads	Mean length (nt)	No. transcripts	Pre-processing	Mapping	FindRGs	Total
Olive tree pistil	767,963	525	9157	24 min 45 s	26 s	5 s	25 min 16 s
				8 nodes, 192 cpus	3 nodes, 72 cpus	1 node, 9 cpus	
*Arabidopsis*	23,821,198 (x2)	100 (x2)	35,386	43 s	19 s	0.2 s	1 min 2 s
				8 nodes, 192 cpus	2 nodes, 48 cpus	1 node, 9 cpus	
Human prostate	969,884,666 (x2)	90 (x2)	176,241	28 s	2 min 37 s	0.03 s	3 min 5 s
				96 nodes, 2304 cpus	24 nodes, 576 cpus	1 node, 9 cpus	

All time values are referred to 100,000 reads when executed on SUSE^®^ Linux Enterprise Server v12 using Opteron processors with 4 GB/core of RAM

### Candidate RGs in reproductive tissues of olive tree

**Fig. 2:**
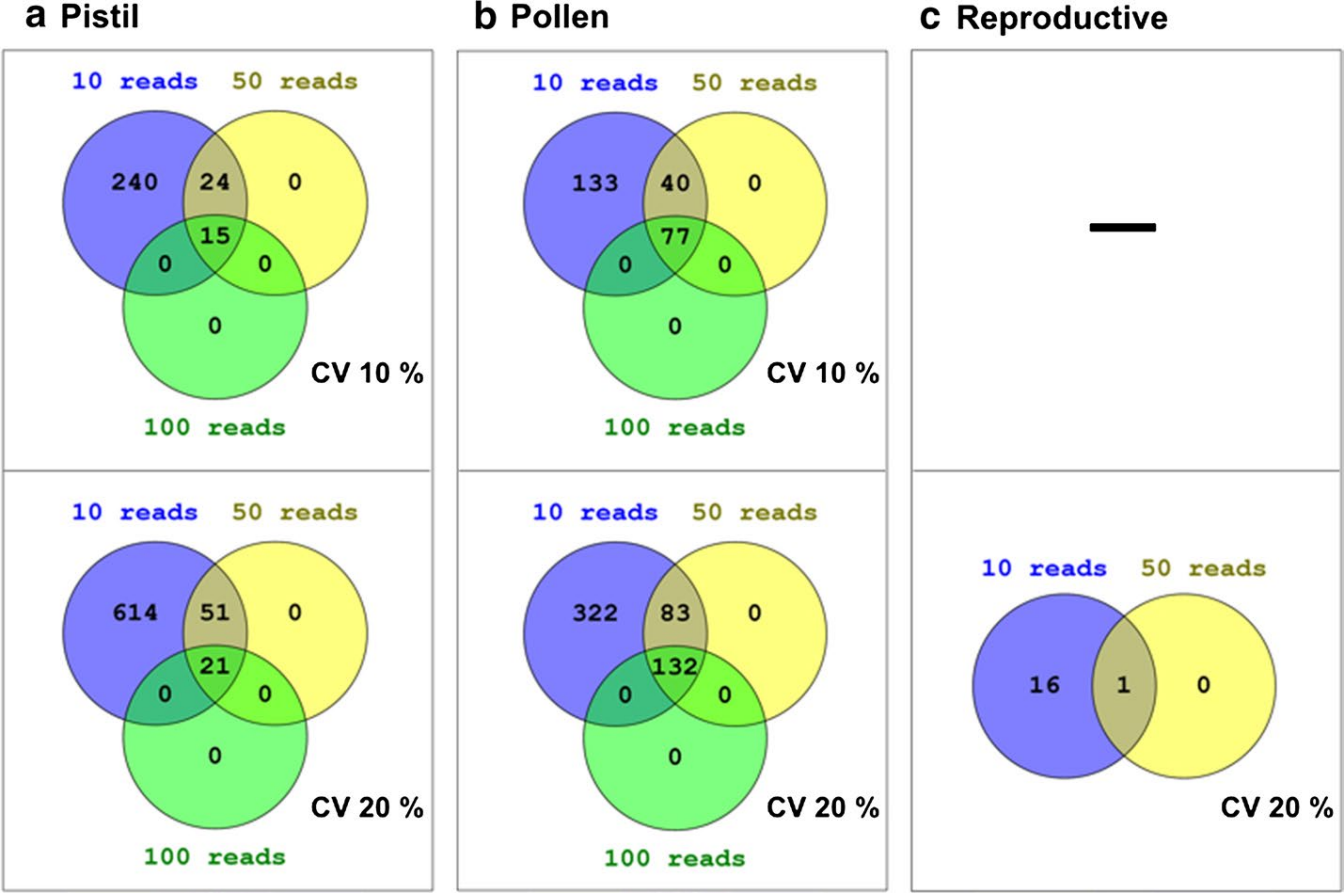
Venn diagrams summarizing the number of RGs obtained for reproductive tissues of olive tree. Two cut-off values were used for CV and three for counted reads. Reproductive RGs were obtained after combining both pollen and pistil reads

Some transcripts are better suited RGs for the analysis of gene expression within a given tissue. Moreover, some of these RGs can even be considered appropriate for gene expression analyses involving several tissues [37]. For this reason, three different executions of the workflow were made in olive: pollen, pistil and both together. Less variant transcripts were retained with two CV cut-off values: 10% (default) and 20% (non-stringent). Taking into account that reads come from Roche/454 platform, the minimum number of mapped reads per gene was set to 10, 50 or 100. A comparative summary of results is shown on Fig. 2. While a significant number of candidates are obtained for pollen and pistil, respectively, with the most stringent conditions (>100 reads and CV < 10%), no candidate is obtained for both pollen and pistil and just one with less rigorous parameters (>50 reads, CV < 20%). This suggests that comparative expression analyses of reproductive tissues require a careful selection of RGs.

A detailed analysis of RGs obtained for these experimental conditions in olive trees have already been suggested and/or used as RGs in others species. In the pistil of the olive tree (Fig. 2a), one of the best RG is ubiquitin (with three different sequences: rp11_olive_006479, rp11_olive_031243, rp11_olive_045557; Additional file 2). Ubiquitin is a traditional and extensively used RG in plants, for example, in banana [38], peach [39] and rice [40], among others. Polyubiquitin 10 (rp11_olive_006473; Additional file 2) also appears as RG and its highly stable expression has been also proven in *Arabidopsis* [15]. It has been validated as an RG in blueberry [41], cotton [42] and poplar [14], and used for normalizing in a work regarding olive fruit development and ripening [43]. Elongation factor 1−α (two different sequences: rp11_olive_008243 and rp11_olive_009319; Additional file 2) also emerges as a candidate RG in the olive pistil. It was evaluated as candidate for RG in potato (*Solanum tuberosum*), resulting in the most stable among the group tested during biotic and abiotic stresses [44]. It is therefore suggested that pistil is another organ where this gene is stable. It has also been validated as a good RG in many species. Another candidate is glyceraldehyde-3-phosphate dehydrogenase (rp11_olive_003751; Additional file 2), which was identified as one of the best RGs for olive fruit development and ripening [20] and used as normalizer for the analysis of cDNAs associated with alternate bearing in olive [45]. Other candidates obtained in olive pistil have never been used before as RGs, but they show outstanding RPMM values and very low CV. This is the case, among others, of salicylic acid-binding protein 2 (rp11_olive_003751) and methylesterase 1 (rp11_olive_015883). Their use should be carefully considered and evaluated in the near future.

A larger number of candidate RGs are suggested for olive pollen (Fig. 2b; Additional file 3). No evidence is present in literature about their use as RGs for most of them, in spite of their apparently low variation in their expression. This is the case of MOB kinase activator (rp11_olive_000239; Additional file 3) or cytochrome P450 (rp11_olive_006957; Additional file 3). Their effectiveness as RGs merits the testing. The only gene proposed by the workflow as suitable RG for olive pollen that has been previously used is cysteine proteinase (rp11_olive_005653; Additional file 3). This gene has been validated as stable and as a suitable RG in *Coffea arabica* [46]. It was also evaluated as RG in olive fruit, but it was not among the most stable tested genes [21], at least in this tissue.

For both pollen and pistil tissues (“Reproductive” in Fig. 2c), two recognized RGs have been proposed in our analysis (Table 3). One of them, *S*-adenosylmethionine decarboxylase (rp11_olive_005197_split_1), was previously pointed out as one of the most abundant sequence in expressed sequence tag (ESTs) libraries of potato (*Solanum tuberosum*) [47]. In fact, this gene also emerged as candidate RG in pollen (Additional file 3) and pistil (Additional file 2). On the other hand, actin 7 (rp11_olive_005099; Table 3) has been extensively employed as RG in many species, such as chicory [48], berry [49] and pea [50]. Likewise, actin 7 also appears as a RG in the pollen analysis (Additional file 3) and it can also be observed in pistil under slightly less restrictive conditions (>50 reads, CV < 10%; results non shown). Once again, unknown candidate RGs are obtained. Shaggy-related protein kinase eta (rp11_olive_006695; Table 3), for instance, would be another interesting gene to test. We can conclude that the approach followed here for reproductive tissues, alone or in combination, yields a set of RGs which is widely supported by previous results described in the literature.

**Table 3:** Best RGs in reproductive tissues (combination of pollen and pistil) of olive tree according to Fig. 2c and ranked by CV

Transcript_id	RPMM						CV (%)	Mean RPMM	Best hit	Description
	PM	PG1	PG5	S2	S3	S4				
rp11_olive_006695	205	209	206	208	177	154	10.72	193.2	Q39011	Shaggy-related protein kinase eta *Arabidopsis thaliana*
rp11_olive_000781	105	140	112	104	108	127	11.36	116	Q94A41	Alpha-amylase 3, chloroplastic *Arabidopsis thaliana*
rp11_olive_006061	327	272	238	343	285	253	13.16	286.3	Q8VZ80	Polyol transporter 5 *Arabidopsis thaliana*
rp11_olive_006091	283	221	211	208	177	190	15.65	215	A0A022R151	Uncharacterized protein *Erythranthe guttata*
rp11_olive_010107	228	213	184	250	295	199	15.98	228.2	O23254	Serine hydroxymethyltransferase 4 *Arabidopsis thaliana*
rp11_olive_005197_split_1	366	430	381	343	423	552	16.37	415.8	Q42679	S-adenosylmethionine decarboxylase proenzyme *Catharanthus roseus*
rp11_olive_003279	122	179	153	114	118	145	16.64	138.5	Q9LV37	Mitogen-activated protein kinase 9 *Arabidopsis thaliana*
rp11_olive_000623	94	94	58	104	108	100	17.68	93	A0A068V6W8	Coffea canephora DH200 = 94 genomic scaffold, scaffold_132 *Coffea canephora*
rp11_olive_007981	144	149	108	156	147	91	18.2	132.5	Q93Y40	Oxysterol-binding protein-related protein 3C *Arabidopsis thaliana*
rp11_olive_005099	888	728	678	530	550	579	18.84	658.8	P53492	Actin-7 *Arabidopsis thaliana*
rp11_olive_005815	311	272	256	322	364	444	19.03	328.2	P17598	Catalase isozyme 1 *Gossypium hirsutum*
rp11_olive_000209_split_1	161	115	117	166	118	100	19.16	129.5	Q67YI9-2	2 of Clathrin interactor EPSIN 2 *Arabidopsis thaliana*
rp11_olive_001245	161	175	108	166	187	118	19.16	152.5	A5A7I7	Calcium-dependent protein kinase 4 *Solanum tuberosum*
rp11_olive_008079	239	204	197	343	275	263	19.34	253.5	M1AVD3	Uncharacterized protein *Solanum tuberosum*
rp11_olive_008883	128	119	144	187	108	118	19.51	134	Q9LZI2	UDP-glucuronic acid decarboxylase 2 *Arabidopsis thaliana*
rp11_olive_035033	178	166	224	177	285	199	19.76	204.8	P62201	Calmodulin *Lilium longiflorum*
rp11_olive_029725	211	128	184	177	157	118	19.82	162.5	O04834	GTP-binding protein SAR1A *Arabidopsis thaliana*

They were obtained for different stages of pollen and pistil with CV < 20% and minimum counted reads of 10. *Transcript_id*: transcript identifiers in the ReprOlive transcriptome

Since RG candidates for reproductive tissues were only obtained when less stringent parameters than in pollen or pistil separately were used (Fig. 2), special care should be taken with such reproductive candidates to RGs. Moreover, these differences may reflect substantial differences in the differentiation of both tissues, in such a way that nearly none gene has the same expression level in both tissues.

### Experimental validation of RGs in olive tree

**Fig. 3:**
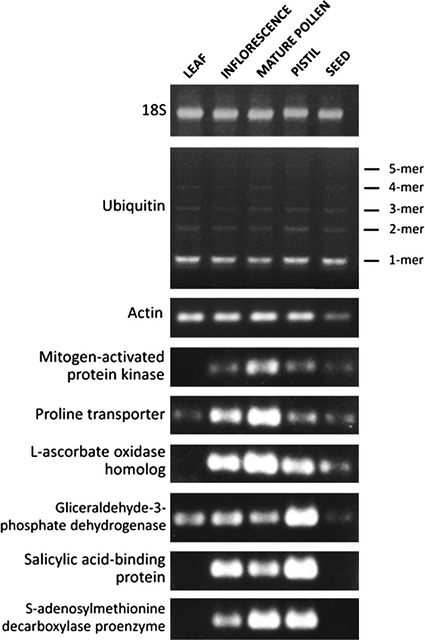
Preliminary RT-PCR validation of RGs predicted in this work in olive tissues in comparison to 18S

It can be thought that the number of reads in a 454/Roche sequencing experiment is not enough to obtain a reliable prediction of RGs. Therefore, an experimental validation was envisaged to further support the predicted RGs obtained with *findRGs*. Polyubiquitin and actin were validated by RT-PCR in different olive tissues in comparison to 18S, a widely used RG (Fig. 3). Both genes show thick and similar expression levels in reproductive tissues (mature pollen and pistil). However, while ubiquitin seems to be a good RG in pollen and pistil, as well as in inflorescences, leafs and seeds, actin was not a good RG for seed. The other commented RGs in previous section resulted in the following outcomes: Two of the transcripts with lower variation in mature pollen (rp11_olive_002359: Mitogen-activated protein kinase, and rp11_olive_009589: Proline transporter 2) showed a good level of expression in both the mature pollen and the whole olive inflorescence, however, they presented lower/null expression in the pistil, seed and leaf. As expected, RT-PCR amplification of rp11_olive_004773, l-ascorbate oxidase homolog (one of the most expressed transcripts in olive pollen), also displayed a similar pattern, with bands of very high intensity corresponding to both the mature pollen and the inflorescence. Regarding transcripts proposed as RGs for the olive pistil due to their low variation, testing of the transcript rp11_olive_003751, glyceraldehyde-3-phosphate dehydrogenase, by RT-PCR resulted in high expression in the pistil, and lower expression in the remaining tissues, including vegetative tissues as those of the leaf. The highly expressed transcript in the pistil rp11_olive_019507, salicylic acid-binding protein 2, generated an intense amplification band in the pistil, the mature pollen and the whole inflorescence, with no amplification in vegetative/seed tissues. Finally, a similar pattern of expression was detected when the proposed RG for reproductive (pollen + pistil) tissues was validated by RT-PCR. In this case, bands of identical intensity were present in both the pollen and the pistil, and with lower intensity, in the whole inflorescence. Overall, RT-PCR validations showed a high degree of consistency with the results obtained by bioinformatics methods, even though a limited number of long reads were obtained by 454/Roche platform. This finding suggests that our bioinformatic approach should be widely used before any RT-PCR or qPCR experiment is carried out. In conclusion, the preliminary RT-PCR validation of the predicted RGs provides reliability to the *findRGs* workflow approach.

### Candidate RGs in *Arabidopsis thaliana*

**Fig. 4:**
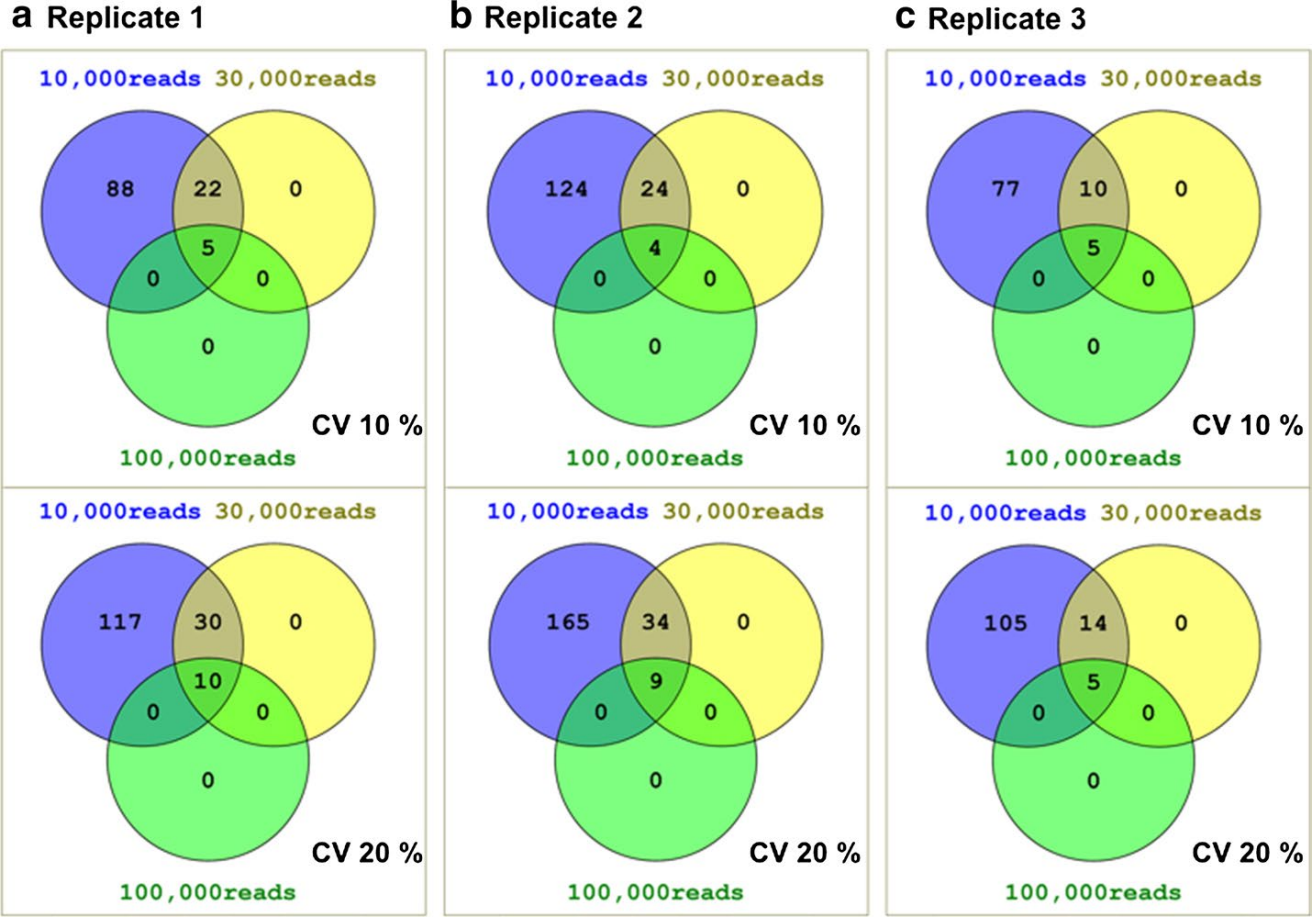
Venn diagrams summarizing the number of RGs obtained for *Arabidopsis thaliana*. Two cut-off values were used for CV and three for counted reads

The successful use of *findRGs* in olive tree drove us to extend it use to more complex datasets. Using publicly available SRA data in PRJEB9470 from *Arabidopsis*, three executions (per replicate) of the workflows in Fig. 1 were carried out (Fig. 4). As before, less variant transcripts were retained with two CV cut-off values: 10% (default) and 20% (non-stringent), but, since these reads were obtained with the Illumina platform, a greater minimum value of counted reads per gene is required. This minimum was set to 10,000, 30,000, and 100,000 for comparative reasons. The lower number of candidates shown in Fig. 4 with respect to Fig. 2 can be explained by the different count threshold due to the different sequencing technology. The number of candidate RGs with the most stringent conditions (>100,000 reads, CV < 10%) is very homogeneous for the three replicates (Fig. 4a–c) and they refer almost exclusively to the same gene, ribulose-1,5-bisphosphate carboxylase/oxygenase (rubisco) (AT1G67090, AT5G38410, AT5G38420, AT5G38430; Table 4), being by far the best candidate in all the cases since their RPMM is much greater than other candidate RGs emerging in less stringent conditions (Additional file 4). Rubisco has been previously used as RG in tea leaf tissues [51]. However, it is not a good candidate for non-green (non-photosynthetic) tissues like those of the anther, neither for pollen [52].

**Table 4:** Best RGs in *Arabidopsis thaliana* according to Fig. 4 and ranked by CV

*Arabidopsis*	transcript_id	RPMM		CV (%)	Mean RPMM	Description
		Col_0	Kil_0			
Replicate 1	AT1G67090.1	9476	9191	1.53	9333.5	Ribulose bisphosphate carboxylase small chain 1A
	AT5G38410.1	7760	8135	2.36	7947.5	Ribulose bisphosphate carboxylase (small chain) family protein
	AT5G38430.1	7054	7548	3.38	7301	Ribulose bisphosphate carboxylase (small chain) family protein
	AT2G39730.1	4051	4343	3.48	4197	Rubisco activase
	AT5G38420.1	7329	7889	3.68	7609	Ribulose bisphosphate carboxylase (small chain) family protein
Replicate 2	AT2G39730.1	3906	4323	5.07	4114.5	Rubisco activase
	AT1G67090.1	8523	9636	6.13	9079.5	ribulose bisphosphate carboxylase small chain 1A
	AT1G21310.1	7013	7976	6.42	7494.5	Extensin 3
	AT5G38410.1	7047	8438	8.98	7742.5	Ribulose bisphosphate carboxylase (small chain) family protein
Replicate 3	AT5G38420.1	8708	8526	1.06	8617	Ribulose bisphosphate carboxylase (small chain) family protein
	AT5G38430.1	8424	8169	1.54	8296.5	Ribulose bisphosphate carboxylase (small chain) family protein
	AT2G39730.1	4365	4524	1.79	4444.5	Rubisco activase
	AT5G38410.1	9172	8822	1.95	8997	Ribulose bisphosphate carboxylase (small chain) family protein
	AT1G67090.1	11,051	9694	6.54	10,372.5	Ribulose bisphosphate carboxylase small chain 1A

They were obtained for the three replicates with CV < 10% and minimum counted reads of 100,000. *Transcript_id*: transcript identifiers in TAIR database

Making a comparison between candidate RGs obtained with less rigorous conditions (>10,000 reads and CV < 10%), 77 candidates are shared by the three replicates (Additional file 4). Several of these genes have been previously commented in this paper as widespread used RGs, such as glyceraldehyde-3-phosphate dehydrogenase (AT3G26650, AT1G42970, AT3G04120 and AT1G13440; Additional file 4), and *S*-adenosylmethionine decarboxylase (AT3G02470; Additional file 4). Additionally, other RGs commonly used in literature emerged, such as phosphoglycerate kinase 1 (AT3G12780; Additional file 4), typically used as control. It shows constant expression levels in leaves, fruit and flowers in tomato [37] and it has also been described as one of the best RGs in *Chrysanthemum* species subjected to different kind of stresses [53]. Several ribosomal proteins are within the candidates (AT1G43170, AT1G02780, AT3G25520, AT5G39740, AT1G56070 and AT5G20290; Additional file 4). They are listed as housekeeping genes and have been suggested as RGs based on analysis of microarray data [54]. However, since all these genes have significant expression variation across tissues, their suitability should be tested in every particular situation. α-Tubulin (AT1G50010 and AT1G04820; Additional file 4) also emerged as candidate. Although it has been extensively used as RG, controversial data have been reported on its reliability, being considered the best in certain species and the worst one in others [20]. There are many others well positioned candidate RGs emerging in all executions that would deserve experimental testing, for instance fructose-bisphosphate aldolase (AT2G21330 and AT4G38970; Additional file 4) or GTP binding elongation factor Tu (AT1G07920, AT1G07930, AT1G07940 and AT5G60390; Additional file 4).

The fact that the same gene appears as candidate RG in the three replicates separately indicates that the possible variability between replicates appears not to be affecting the estimation. The calculation of RGs combining all replicates extracted the same candidate RGs (results not shown), suggesting that an average number of reads per replicate of ~11,000,000 (Additional file 1) could be enough for the aim of the workflow. In conclusion, the list of candidate RGs obtained by means of our workflow offers a first and reliable estimation of the most appropriate RGs for expression studies between these two *Arabidopsis* strains in these particular experimental conditions. It also suggests that mapping with less than 11 millions of reads could be enough to obtain a reliable prediction.

### RGs and human cancers

Many studies on cancer perform multiple comparisons (between tumors and normal tissues, different stages, response to treatments…). RGs needed for these comparisons should have consistent expression level in the conditions to be analyzed. The search of appropriate RGs in such cases becomes particularly tricky and challenging, since cancer is associated with changes in gene expression involving many pathways, and it is demonstrated a huge heterogeneity within and among cancers [55]. Even traditional housekeeping genes are likely to change their expression level during the course of the disease [56], since they might not only be implicated in the basal cell metabolism but also in other cell functions [57]. Therefore, it is crucial to perform preliminary evaluations for identifying the most stably expressed genes in each situation. Moreover, it is not unusual that cancer experiments have many tens of replicates [28]. Therefore, this is a good situation to test if *findRGs* can cope with large amount of samples with sample size heterogeneity in a high throughput experiment. Taking into account the higher number of samples and the possible increase of variability, less stringent filtering parameters values of maximum CV and minimum counted reads were tested and adapted in each particular situation.

### Candidate RGs for prostate cancer

**Fig. 5:**
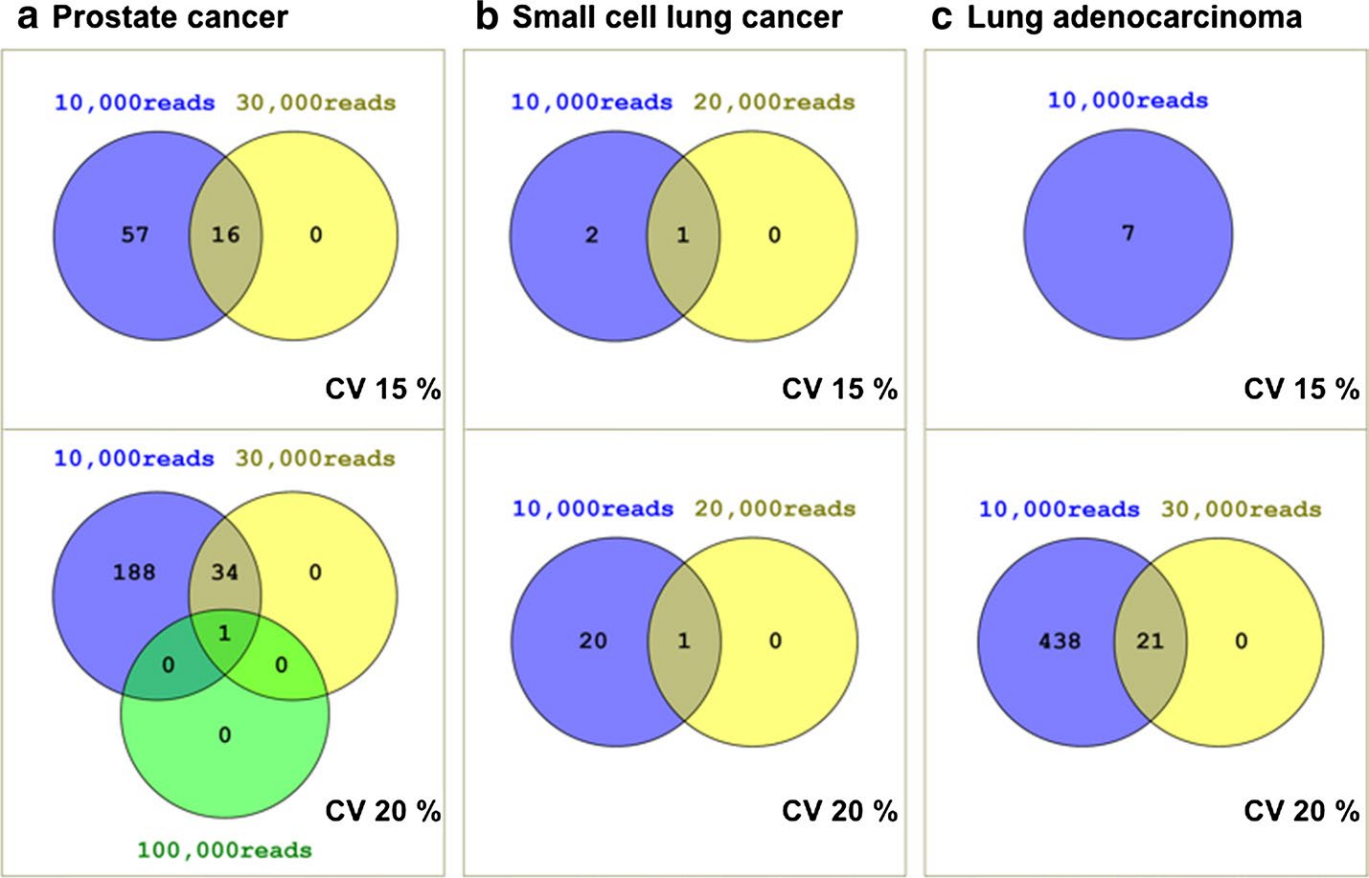
Venn diagrams summarizing the number of RGs obtained for matched samples of normal and malignant tissues of three different human cancers: prostate, small-cell lung cancer and lung adenocarcinoma. Two cut-off values per cancer were used for CV and different counted reads depending on the tissue

Figure 5a shows the number of candidate RGs using different cut-off values for CV and counted reads in prostate samples. No candidate RG is obtained in the most stringent conditions (>100,000 and CV < 15%) and only one with a slightly more permissive maximum CV value of 20%. More RGs were obtained using less stringent conditions; those obtained with >30,000 and CV < 20% are presented in Table 5. Many of them have been used as RGs in cancer studies. For example, the tyrosine 3-monooxygenase/tryptophan 5-monooxygenase activation protein zeta polypeptide (ENST00000395957.6; Table 5) has been repeatedly studied as RG candidate in prostate cancer, although it has not been between the most stable genes [58, 59]. Nevertheless, it is one of the best ranked as stable genes for the comparison between cancer stem cells and native cells [60]. Nascent-polypeptide-associated complex alpha polypeptide (ENST00000356769.7; Table 5) is a human housekeeping gene evaluated and sometimes proposed as RG in several types of cancer, such as breast cancer [61] or colon cancer [11]. Phosphoglycerate kinase 1 (ENST00000373316.4; Table 5), another human housekeeping gene, was typically used as RG, as in plants, and has been demonstrated to be affected between normal and malignant tissues in certain malignancies, but not in others [60, 62]. Genes encoding the different subunits of ATP synthase mitochondrial are considered human housekeeping genes [63], some of them considered RGs for some tumors [11], whereas no in all [64]. Following our results, ENST00000398752.10 and ENST00000495596.5 (Table 5) should merit experimental consideration. Several ribosomal proteins are also proposed as RGs (ENST00000314138.10, ENST00000519807.5, ENST00000338970.10 and ENST00000456530.6; Table 5). Despite their widely spread use as RGs, expression variations of these genes have been detected not only between tumors and healthy tissues [65], but also across normal tissues [54].

**Table 5:** Best candidate RGs for normal and malignant prostate tissues according to Fig. 5a and ranked by CV

Transcript_id	CV (%)	Mean RPMM	Gene	Description
ENST00000510199.5	8.95	99.8	GNB2L1	Guanine nucleotide binding protein (G protein), beta polypeptide 2-like 1
ENST00000425566.1	9.91	127.3	RPL23AP87	Ribosomal protein L23a pseudogene 87
ENST00000314138.10	10.52	134.9	RPL27A	Ribosomal protein L27a
ENST00000412331.6	11.18	108.1	EIF3L	Eukaryotic translation initiation factor 3 subunit L
ENST00000494591.1	11.49	78.4	RPSAP36	Ribosomal protein SA pseudogene 36
ENST00000519807.5	11.5	168.1	RPS20	Ribosomal protein S20
ENST00000356769.7	11.58	92.8	NACA	Nascent polypeptide-associated complex alpha subunit
ENST00000496593.5	12.28	253.5	RPLP0P2	Ribosomal protein, large, P0 pseudogene 2
ENST00000338970.10	12.63	176.9	RPL14	Ribosomal protein L14
ENST00000610672.4	12.74	244	MED22	Mediator complex subunit 22
ENST00000395957.6	12.97	95.5	YWHAZ	Tyrosine 3-monooxygenase/tryptophan 5-monooxygenase activation protein, zeta
ENST00000234831.9	13.69	108.1	TMEM59	Transmembrane protein 59
ENST00000353047.10	14.01	156.1	CTSB	Cathepsin B
ENST00000556083.1	14.36	137.5	ACTN1	Actinin, alpha 1
ENST00000558264.5	14.59	129.8	TPM1	Tropomyosin 1 (alpha)
ENST00000394621.6	14.87	189.2	STEAP2	STEAP2 metalloreductase
ENST00000335508.10	15.15	116	SF3B1	Splicing factor 3b subunit 1
ENST00000341423.9	15.26	134.4	HMGB1	High mobility group box 1
ENST00000564521.6	15.84	167	ALDOA	Aldolase, fructose-bisphosphate A
ENST00000398752.10	16.45	200.6	ATP5A1	ATP synthase, H + transporting, mitochondrial F1 complex, alpha subunit 1, cardiac muscle
ENST00000264657.9	16.84	137.7	STAT3	Signal transducer and activator of transcription 3 (acute-phase response factor)
ENST00000357214.5	17.4	105.5	SFPQ	Splicing factor proline/glutamine-rich
ENST00000456530.6	17.44	118.5	RPL15	Ribosomal protein L15
ENST00000495596.5	17.64	164.2	ATP5G2	ATP synthase, H + transporting, mitochondrial Fo complex subunit C2 (subunit 9)
ENST00000391959.5	17.67	125.6	PPP1R12B	protein phosphatase 1 regulatory subunit 12B
ENST00000369936.2	17.97	235	KIAA1324	KIAA1324
ENST00000300619.11	19.01	104.5	ZNF91	Zinc finger protein 91
ENST00000401722.7	19.21	156.4	SLC25A3	Solute carrier family 25 (mitochondrial carrier; phosphate carrier), member 3
ENST00000618621.4	19.43	405.9	LPP	LIM domain containing preferred translocation partner in lipoma
ENST00000249822.8	19.53	107.3	ARPP19	cAMP regulated phosphoprotein 19 kDa
ENST00000353411.10	19.68	122.8	SKP1	S-phase kinase-associated protein 1
ENST00000375856.4	19.76	151.8	IRS2	Insulin receptor substrate 2
ENST00000373316.4	19.79	118.1	PGK1	Phosphoglycerate kinase 1
ENST00000306085.10	19.9	159.2	TRIM56	Tripartite motif containing 56
ENST00000357308.8	20	105	GFPT1	Glutamine–fructose-6-phosphate transaminase 1

They were obtained with CV < 20% and minimum counted reads of 30,000. *Transcript_id*: human transcript identifiers in ENSEMBL database

### Candidate RGs for small-cell lung cancer

No candidates were obtained with the same cut-offs of prostate cancer. Then, RG selection was carried out with minimum number of mapped reads set to 10,000 and 20,000 and CV cut-off values 15 and 20% (Fig. 5b). Even so, the number of candidates with the most stringent CV cut-off (<15%) was very low, indicating variability between samples. The candidate RGs obtained with the less stringent combination of filtering parameters (>10,000 and CV < 20%) are given in Table 6. Ribosomal proteins (ENST00000456530.6, ENST00000422514.6 and ENST00000338970.10; Table 6) and a gene encoding a subunit of ATP synthase mitochondrial (ENST00000495596.5; Table 6) are present, as in prostate. Ubiquitin A-52 residue ribosomal protein fusion product 1 (ENST00000442744.6; Table 6) is also suggested and has been identified as RG in breast cancer [61], as well as in bladder or testis through microarray meta-analysis of human clinical samples [66]. Therefore, these less stringent cut-offs are providing reliable RGs.

**Table 6:** Best candidate RGs for normal lung and small-cell lung cancer according to Fig. 5b and ranked by CV

Transcript_id	CV (%)	Mean RPMM	Gene	Description
ENST00000425566.1	12.68	76.2	RPL23AP87	Ribosomal protein L23a pseudogene 87
ENST00000338970.10	12.96	103.3	RPL14	Ribosomal protein L14
ENST00000442744.6	13.28	69.4	UBA52	Ubiquitin A-52 residue ribosomal protein fusion product 1
ENST00000456530.6	16.02	76.7	RPL15	Ribosomal protein L15
ENST00000553521.5	16.21	50.2	SRSF5	Serine/arginine-rich splicing factor 5
ENST00000373242.6	16.8	73	SAR1A	Secretion associated, Ras related GTPase 1A
ENST00000261890.6	16.88	55.3	RAB11A	RAB11A, member RAS oncogene family
ENST00000510199.5	17.11	66	GNB2L1	Guanine nucleotide binding protein (G protein), beta polypeptide 2-like 1
ENST00000234115.10	17.59	63.6	PLEKHB2	Pleckstrin homology domain containing B2
ENST00000401722.7	17.69	83.6	SLC25A3	Solute carrier family 25 (mitochondrial carrier; phosphate carrier), member 3
ENST00000412331.6	17.76	54.6	EIF3L	Eukaryotic translation initiation factor 3 subunit L
ENST00000422514.6	18.83	80.3	RPL23A	Ribosomal protein L23a
ENST00000342374.4	19.13	45.2	SERINC3	Serine incorporator 3
ENST00000483316.1	19.26	77.6	BAZ2B	Bromodomain adjacent to zinc finger domain 2B
ENST00000335508.10	19.41	72.4	SF3B1	Splicing factor 3b subunit 1
ENST00000471227.3	19.62	66.4	RPL23AP2	Ribosomal protein L23a pseudogene 2
ENST00000334256.8	19.77	46.9	KPNA4	Karyopherin alpha 4 (importin alpha 3)
ENST00000332361.5	19.79	64.5	RPL23AP57	Ribosomal protein L23a pseudogene 57
ENST00000416139.1	19.81	64.5	RPL23AP18	Ribosomal protein L23a pseudogene 18
ENST00000495596.5	19.84	71.5	ATP5G2	ATP synthase, H + transporting, mitochondrial Fo complex subunit C2 (subunit 9)
ENST00000446445.1	19.87	64.1	RPL23AP43	Ribosomal protein L23a pseudogene 43

They were obtained with CV < 20% and minimum counted reads of 10,000. *Transcript_id*: human transcript identifiers in ENSEMBL database

### Candidate RGs for lung adenocarcinoma

Figure 5c shows that samples of normal lung and lung adenocarcinoma are the more variant instances analyzed in this work since only 7 RGs are obtained using >10,000 mapped reads and CV < 15%. Therefore the list of candidate RGs was obtained with a minimum counted reads of 30,000 and a CV cut-off of 20% (Table 7). Some of the RGs (ENST00000270460.10, ENST00000323443.6, ENST00000367975.6, ENST00000528973.1, ENST00000262160.10, ENST00000398004.3, ENST00000396444.7, ENST00000258711.7, ENST00000329627.11 and ENST00000238831.8; Table 7) have been described as human housekeeping genes [63], but there are no evidence about their use as RGs. Several zinc finger proteins (ENST00000328654.9, ENST00000307635.3 and ENST00000253115.6; Table 7) have already been suggested as RGs in cancerous kidney and lymph node tissues [66], but are not suitable RGs for normal and colorectal cancer tissues [64]; however, according our results, they seem to be appropriate for studies in normal and cancerous lung. Some of the candidate RGs previously commented for prostate or small-cell lung cancer, such as ribosomal proteins (Tables 4, 5), are also retained for lung adenocarcinoma with the less stringent conditions (>10,000 reads and CV < 20%; non shown results). This prompted us to think that those transcripts could finally be suitable for studies involving several types of normal and cancerous cells.

**Table 7:** Best candidate RGs for normal normal lung and lung adenocarcinoma according to Fig. 5c and ranked by CV

Transcript_id	CV (%)	Mean RPMM	Gene	Description
ENST00000411857.2	16.34	224.7	HNRNPA1P54	Heterogeneous nuclear ribonucleoprotein A1 pseudogene 54
ENST00000270460.10	18.06	204.1	EPN1	Epsin 1
ENST00000373191.8	18.17	195.4	AGO3	Argonaute 3, RISC catalytic component
ENST00000323443.6	18.2	218.4	LRRC57	Leucine rich repeat containing 57
ENST00000367975.6	18.35	204.8	SDHC	Succinate dehydrogenase complex subunit C
ENST00000528973.1	18.42	211	PCSK7	Proprotein convertase subtilisin/kexin type 7
ENST00000262160.10	18.7	214	SMAD2	SMAD family member 2
ENST00000607772.5	18.73	200.3	CNKSR3	CNKSR family member 3
ENST00000261854.9	18.85	198.2	SPPL2A	Signal peptide peptidase like 2A
ENST00000398004.3	19.12	316.1	SLC35E3	Solute carrier family 35 member E3
ENST00000396444.7	19.21	294	USP8	Ubiquitin specific peptidase 8
ENST00000304177.9	19.28	212.4	C15orf40	Chromosome 15 open reading frame 40
ENST00000328654.9	19.31	241.8	ZNF26	Zinc finger protein 26
ENST00000307635.3	19.34	218.1	ZNF556	Zinc finger protein 556
ENST00000258711.7	19.38	323.7	CHST12	Carbohydrate (chondroitin 4) sulfotransferase 12
ENST00000329627.11	19.41	318.1	PEX26	Peroxisomal biogenesis factor 26
ENST00000322122.7	19.49	192.7	TRIM72	Tripartite motif containing 72, E3 ubiquitin protein ligase
ENST00000238831.8	19.5	291.1	YIPF4	Yip1 domain family member 4
ENST00000258149.9	19.71	222.8	MDM2	MDM2 proto-oncogene, E3 ubiquitin protein ligase
ENST00000253115.6	19.72	227.3	ZNF426	Zinc finger protein 426
ENST00000614987.4	19.74	346.8	RPS6KA5	Ribosomal protein S6 kinase, 90 kDa, polypeptide 5

They were obtained with CV < 20% and minimum counted reads of 30,000. *Transcript_id*: human transcript identifiers in ENSEMBL database

### Candidate RGs for different combination of malignant and normal tissues

**Fig. 6:**
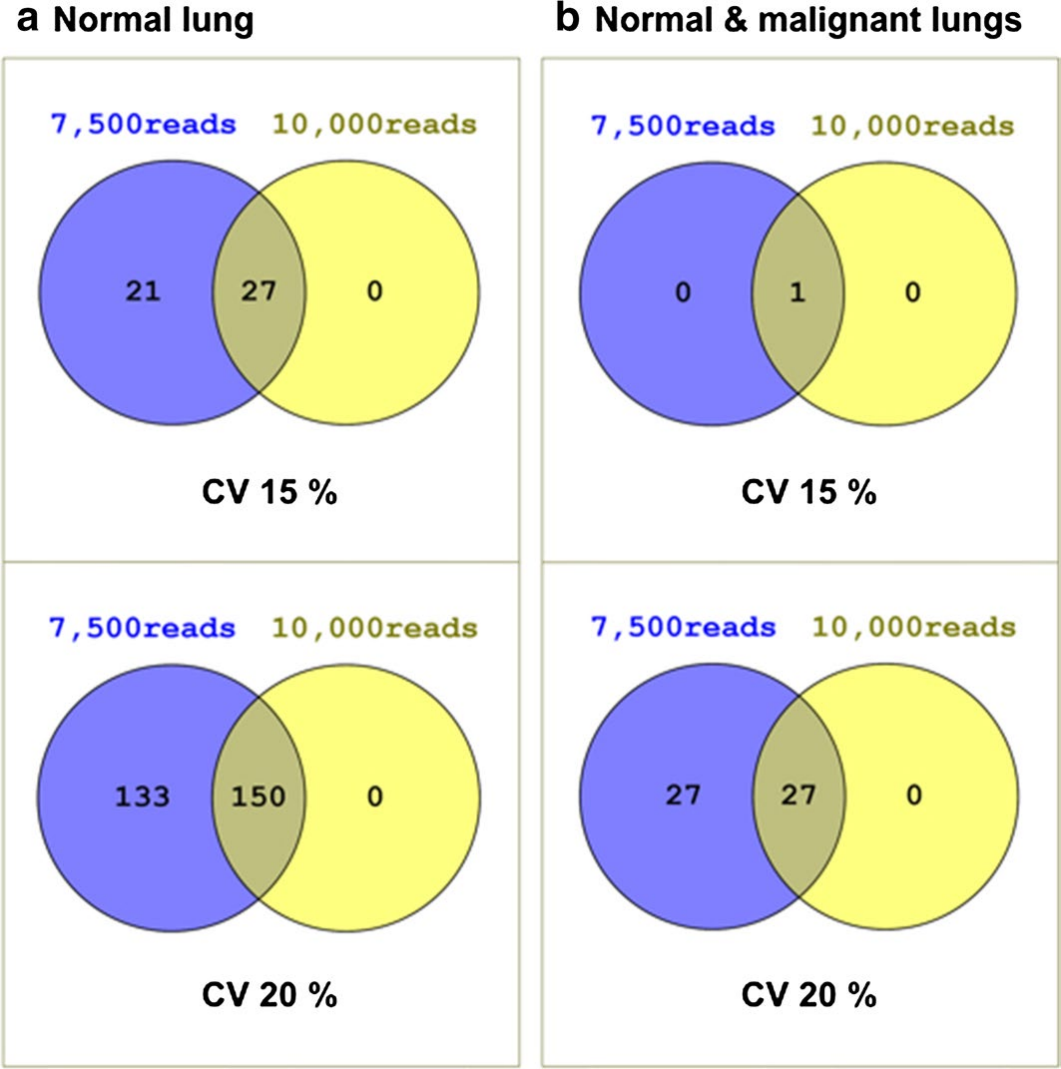
Venn diagrams summarizing the number of RGs obtained for different combinations of lung samples: samples from only normal lung (**a**) or normal and malignant lung (**b**) were analyzed with two CV cut-off values per combination and two different counted reads

Although every cancer is an independent scenario that should be analyzed separately in the search of RGs, different combinations or tissues and/or states were tested. When combining normal and malignant lung samples, filtering parameters were still less stringent: CV is maintained in 15 and 20% and the minimum counted reads was set to 7500 and 10,000 (Fig. 6). The analysis was performed with only normal lung and combining normal and malignant samples. The resulting RG candidates for normal lung are listed in Additional file 5. The resulting RGs for combined normal and malignant lung samples are listed in Additional file 6.

Candidate RGs for normal lung tissues revealed some known RGs, such as nascent-polypeptide-associated complex alpha polypeptide (ENST00000356769.7; Additional file 5) [11, 61], ornithine decarboxylase antizyme 1 (ENST00000586054.2; Additional file 5) [66], although with some up-regulation in cancer [67], and small ubiquitin-like modifier 2 gene [66]. For the whole set of lung samples (Fig. 6b; Additional file 6), the ribosomal protein L14 (ENST00000338970.10; Additional files 5, 6), already described in normal and cancerous kidney tissues [66], was found. Interestingly, ATP synthase subunit alpha mitochondrial (ENST00000398752.10; Additional files 5, 6) has not been described as RG in literature and deserves a careful testing.

The analysis combining the whole normal and malignant human cancers samples described here (prostate cancer, small-cell cancer lung and lung adenocarcinoma) did not provide any RG, revealing that the addition of a completely different tissue (prostate) supposes a new source of variability between samples.

## Conclusion

The automatic workflow presented in this work takes advantage of new and publicly available NGS data to predict the suitable RGs for every experimental situation. Those candidate RGs can be useful for qPCR validation in further expression analyses. The analysis is particularly suitable in non-model species, for which few or no RGs have been identified, or in new experimental conditions where no previous data are available. The workflow seems to be independent of the sequencing technology that generates the reads, the number of reads, as well as the read length, since it seems to work equally well with many short reads (Illumina from *Arabidopsis* and human) than with a few long reads (Roche/454 from olive tree). It supports massive data analyses with low (*Arabidopsis*) and high (adenocarcinoma) number of samples. Time executions for the different tasks of the workflow are reasonably short, since the time consuming parts (pre-processing and mapping) are required for any NGS analysis and are performed only once. Our workflow is customizable and adaptable to the requirements of each experimental case. The algorithm in *findRGs* has been acceptably tested for three species (olive, *Arabidopsis* and human) in comparison studies focused in very different biological aspects, so as different developmental, physiological or pathological stages (reproductive tissues, flower and cancer). Lists of candidate RGs have been generated in every case, some of which have even been already described in the literature and others have been preliminarily validated here (Fig. 3); both findings are supporting this experimental approach. More interestingly, new and more suitable RGs can be discovered with *findRGs*. Even though the expression level and stability of these new RGs may require some experimental validation prior to their utilization for normalization, we encourage the utilization of *findRGs* where possible, since it can be quite helpful as a preliminary approximation about the best RG candidates, prior to each single expression experiment.

## Additional files

**Additional file 1.** Statistics related to pre-processing with SeqTrimNext and mapping with Bowtie2 for the different datasets: olive tree libraries, *Arabidopsis* libraries, normal/malignant prostate samples, normal/small-cell cancer lung samples and normal/adenocarcinoma lung samples.

**Additional file 2.** Best RGs in olive tree pistil according to Fig. 2a, ranked by CV. They were obtained for different stages of pistil development with CV < 10% and minimum counted reads of 100. *Transcript_id*: transcript identifiers in the ReprOlive transcriptome.

**Additional file 3.** Best RGs in olive tree pollen according to Fig. 2b, ranked by CV. They were obtained for different stages of pollen development, with CV < 10% and minimum counted reads of 100. *Transcript_id*: transcript identifiers in the ReprOlive transcriptome.

**Additional file 4.** Best candidate RGs in *Arabidopsis thaliana* for replicates 1, 2 and 3 with their respective RPMM values for each replicate. They were obtained with CV < 10% and a minimum counted reads of 10,000. *Transcript_id*: transcript identifiers in TAIR database.

**Additional file 5.** Best candidate RGs for normal lung tissues according to Fig. 6a and ranked by CV. They were obtained with CV < 15% and minimum counted reads of 10,000. *Transcript_id*: human transcript identifiers in ENSEMBL database.

**Additional file 6.** Best candidate RGs for normal and malignant lung samples according to Fig. 6b, ranked by CV. They were obtained with CV < 20% and minimum counted reads of 10,000. *Transcript_id*: human transcript identifiers in ENSEMBL database.
