# Supplementary material for: Automated identification of reference genes based on RNA-seq data
Source: Biomed Eng Online. 2017 Aug 18;16(Suppl 1):65. doi: 10.1186/s12938-017-0356-5 (PMC5568602; doi:10.1186/s12938-017-0356-5)
Supplement: Supplementary file 1 — Additional file 1. Statistics related to pre-processing with SeqTrimNext and mapping with Bowtie2 for the different datasets: olive tree libraries, Arabidopsis libraries, normal/malignant prostate samples, normal/small-cell cancer lung samples and normal/adenocarcinoma lung samples. [file 12938_2017_356_MOESM1_ESM.docx]

**Additional File 1: Statistics related to pre-processing with SeqTrimNext and mapping with Bowtie2 for the different datasets:** olive tree libraries, *Arabidopsis* libraries, normal/malignant prostate samples, normal/small-cell cancer lung samples and normal/adenocarcinoma lung samples.

|  |  | **Raw reads** | **Useful reads** | **% Useful reads** | **Mapped reads** | **% Mapping** |
| --- | --- | --- | --- | --- | --- | --- |
| **OLIVE TREE** | **Mean length 385 nt** |  |  |  |  |  |
| **Gene library** | **Tissue** |  |  |  |  |  |
| PM | Pollen | 217,163 | 111,760 | 51.46 | 76,197 | 68.18 |
| PG1 | Pollen | 258,167 | 141,232 | 54.71 | 96,746 | 68.5 |
| PG5 | Pollen | 233,921 | 120,276 | 51.42 | 84,081 | 69.91 |
| S2 | Pistil | 257,813 | 138,077 | 53.56 | 63,030 | 45.65 |
| S3 | Pistil | 247,401 | 141,903 | 57.36 | 65,934 | 46.46 |
| S4 | Pistil | 262,749 | 150,185 | 57.16 | 71,044 | 47.3 |
|  |  |  |  |  |  |  |
| ***Arabidopsis thaliana*** | **Paired-end 100 nt** |  |  |  |  |  |
| **Accession** | **Replicate** |  |  |  |  |  |
| Columbia-0 | Col-0-rep1 | 12,251,921 | 11,861,826 | 96.82 | 11,143,112 | 93.94 |
| Columbia-0 | Col-0-rep2 | 12,297,628 | 11,912,459 | 96.87 | 11,178,500 | 93.84 |
| Columbia-0 | Col-0-rep3 | 13,076,233 | 12,678,437 | 96.96 | 11,988,627 | 94.56 |
| Killean-0 | Kil-0-rep1 | 11,210,913 | 10,914,810 | 97.36 | 7,544,520 | 69.12 |
| Killean-0 | Kil-0-rep2 | 11,523,570 | 11,198,260 | 97.18 | 9,214,640 | 82.29 |
| Killean-0 | Kil-0-rep3 | 9,107,610 | 8,859,088 | 97.27 | 6,945,942 | 78.4 |
|  |  |  |  |  |  |  |
| **PROSTATE** | **Paired-end 90 nt** |  |  |  |  |  |
| **Sample id** | **Tissue** |  |  |  |  |  |
| ERR031028 | Malignant | 33,177,845 | 30,766,171 | 92.73 | 16,407,245 | 53.33 |
| ERR031030 | Malignant | 32,289,266 | 29,628,492 | 91.76 | 19,964,313 | 67.38 |
| ERR031032 | Malignant | 32,319,406 | 29,850,038 | 92.36 | 22,555,415 | 75.56 |
| ERR299297 | Malignant | 34,505,542 | 31,381,770 | 90.95 | 18,661,591 | 59.47 |
| ERR299298 | Malignant | 34,650,697 | 31,519,379 | 90.96 | 21,413,441 | 67.94 |
| ERR031038 | Malignant | 35,679,519 | 32,873,785 | 92.14 | 22,367,251 | 68.04 |
| ERR031040 | Malignant | 33,974,921 | 31,295,136 | 92.11 | 20,482,189 | 65.45 |
| ERR031042 | Malignant | 34,988,865 | 31,422,099 | 89.81 | 23,858,148 | 75.93 |
| ERR031044 | Malignant | 34,758,125 | 30,976,591 | 89.12 | 21,088,203 | 68.08 |
| ERR031018 | Malignant | 34,007,787 | 31,732,742 | 93.31 | 18,923,690 | 59.63 |
| ERR299295 | Malignant | 34,718,521 | 32,023,917 | 92.24 | 18,195,362 | 56.82 |
| ERR031022 | Malignant | 36,820,858 | 33,582,601 | 91.21 | 23,069,772 | 68.7 |
| ERR031024 | Malignant | 37,576,110 | 34,672,572 | 92.27 | 22,138,262 | 63.85 |
| ERR031026 | Malignant | 36,886,097 | 33,106,211 | 89.75 | 24,549,357 | 74.15 |
| ERR031027 | Normal | 36,136,601 | 33,206,257 | 91.89 | 26,027,074 | 78.38 |
| ERR031029 | Normal | 35,534,313 | 31,883,216 | 89.73 | 23,976,174 | 75.2 |
| ERR031031 | Normal | 31,921,622 | 29,131,720 | 91.26 | 22,924,313 | 78.69 |
| ERR031033 | Normal | 33,965,736 | 31,544,427 | 92.87 | 27,449,070 | 87.02 |
| ERR031035 | Normal | 35,569,937 | 32,247,476 | 90.66 | 24,077,166 | 74.66 |
| ERR299299 | Normal | 36,320,661 | 33,509,756 | 92.26 | 27,735,638 | 82.77 |
| ERR031039 | Normal | 38,401,723 | 34,634,503 | 90.19 | 29,374,960 | 84.81 |
| ERR031041 | Normal | 33,191,569 | 30,863,303 | 92.99 | 23,393,960 | 75.8 |
| ERR031043 | Normal | 34,266,043 | 31,501,550 | 91.93 | 23,200,444 | 73.65 |
| ERR031017 | Normal | 34,536,162 | 32,226,681 | 93.31 | 24,914,252 | 77.31 |
| ERR031019 | Normal | 36,250,477 | 33,148,288 | 91.44 | 28,774,650 | 86.81 |
| ERR299296 | Normal | 32,272,887 | 29,741,168 | 92.16 | 22,079,393 | 74.24 |
| ERR031023 | Normal | 31,245,264 | 28,690,318 | 91.82 | 22,622,032 | 78.85 |
| ERR031025 | Normal | 33,918,112 | 31,856,414 | 93.92 | 25,272,051 | 79.33 |
|  |  |  |  |  |  |  |
| **LUNG** | **Paired-end 75 nt** |  |  |  |  |  |
| **Sample id** | **Tissue** |  |  |  |  |  |
| 98687 | Small-cell cancer | 56,036,324 | 48,859,671 | 87.19 | 39,912,273 | 81.69 |
| 134427 | Small-cell cancer | 32,825,305 | 28,526,405 | 86.9 | 24,359,210 | 85.39 |
| 134430 | Small-cell cancer | 32,659,995 | 29,391,152 | 89.99 | 25,180,728 | 85.67 |
| 585210 | Small-cell cancer | 31,124,598 | 27,611,517 | 88.71 | 24,595,833 | 89.08 |
| 585270 | Small-cell cancer | 31,198,761 | 27,116,894 | 86.92 | 23,413,039 | 86.34 |
| 98711 | Small-cell cancer | 38,390,350 | 33,913,898 | 88.34 | 28,027,877 | 82.64 |
| 585260 | Small-cell cancer | 31,014,041 | 27,253,019 | 87.87 | 23,755,451 | 87.17 |
| 585272 | Small-cell cancer | 29,622,714 | 26,420,826 | 89.19 | 21,765,044 | 82.38 |
| 585267 | Small-cell cancer | 39,240,348 | 34,403,714 | 87.67 | 29,236,522 | 84.98 |
| 98735 | Small-cell cancer | 53,174,680 | 47,439,393 | 89.21 | 38,790,717 | 81.77 |
| 631076 | Small-cell cancer | 56,371,020 | 52,374,098 | 92.91 | 41,786,421 | 79.78 |
| 631092 | Small-cell cancer | 68,511,568 | 62,601,354 | 91.37 | 55,434,666 | 88.55 |
| 113368 | Small-cell cancer | 63,785,735 | 55,437,729 | 86.91 | 47,866,417 | 86.34 |
| 134398 | Small-cell cancer | 33,554,527 | 29,421,957 | 87.68 | 26,509,492 | 90.1 |
| 134421 | Small-cell cancer | 27,615,978 | 24,176,609 | 87.55 | 20,618,746 | 85.28 |
| 134413 | Small-cell cancer | 36,313,176 | 32,197,352 | 88.67 | 27,690,789 | 86 |
| 134417 | Small-cell cancer | 22,287,482 | 19,721,315 | 88.49 | 16,235,513 | 82.32 |
| 98691 | Normal | 57,034,457 | 49,797,771 | 87.31 | 44,719,612 | 89.8 |
| 134428 | Normal | 27,072,395 | 24,341,356 | 89.91 | 22,126,837 | 90.9 |
| 134429 | Normal | 29,094,496 | 26,074,176 | 89.62 | 22,207,584 | 85.17 |
| 585216 | Normal | 32,418,652 | 28,639,781 | 88.34 | 25,668,950 | 89.63 |
| 585275 | Normal | 30,063,713 | 26,542,332 | 88.29 | 23,470,905 | 88.43 |
| 98714 | Normal | 40,802,954 | 34,967,706 | 85.7 | 27,561,859 | 78.82 |
| 585266 | Normal | 32,601,483 | 28,699,668 | 88.03 | 25,911,927 | 90.29 |
| 585277 | Normal | 26,150,587 | 21,291,635 | 81.42 | 17,909,717 | 84.12 |
| 585273 | Normal | 23,988,291 | 20,369,400 | 84.91 | 14,191,388 | 69.67 |
| 98739 | Normal | 59,396,738 | 50,983,292 | 85.84 | 43,061,780 | 84.46 |
| 631078 | Normal | 51,507,115 | 47,704,943 | 92.62 | 38,518,942 | 80.74 |
| 631094 | Normal | 51,396,274 | 45,772,389 | 89.06 | 35,572,748 | 77.72 |
| 113370 | Normal | 49,041,887 | 42,354,941 | 86.36 | 34,158,202 | 80.65 |
| 134400 | Normal | 34,723,224 | 30,468,879 | 87.75 | 26,954,002 | 88.46 |
| 134423 | Normal | 26,521,369 | 23,145,770 | 87.27 | 20,736,966 | 89.59 |
| 134416 | Normal | 19,374,213 | 16,828,970 | 86.86 | 14,450,914 | 85.87 |
| 134420 | Normal | 24,739,506 | 21,583,729 | 87.24 | 19,098,298 | 88.48 |
|  |  |  |  |  |  |  |
| **LUNG** | **Paired-end 100 nt** |  |  |  |  |  |
| **Sample id** | **Tissue** |  |  |  |  |  |
| ERR062334 | Adenocarcinoma | 35,853,815 | 27,109,654 | 75.61 | 21,350,668 | 78.76 |
| ERR062335 | Adenocarcinoma | 44,654,797 | 33,478,179 | 74.97 | 25,816,712 | 77.12 |
| ERR062336 | Adenocarcinoma | 52,449,083 | 39,215,337 | 74.77 | 29,702,388 | 75.74 |
| ERR062337 | Adenocarcinoma | 48,421,741 | 35,691,867 | 73.71 | 29,576,462 | 82.87 |
| ERR062338 | Adenocarcinoma | 46,449,533 | 35,043,040 | 75.44 | 26,379,156 | 75.28 |
| ERR164586 | Adenocarcinoma | 35,597,808 | 29,913,857 | 84.03 | 24,277,572 | 81.16 |
| ERR164588 | Adenocarcinoma | 22,541,432 | 18,602,595 | 82.53 | 14,649,548 | 78.75 |
| ERR164589 | Adenocarcinoma | 109,352,960 | 93,361,284 | 85.38 | 74,883,133 | 80.21 |
| ERR164590 | Adenocarcinoma | 25,639,079 | 22,787,631 | 88.88 | 19,328,193 | 84.82 |
| ERR164591 | Adenocarcinoma | 40,642,669 | 35,388,780 | 87.07 | 27,701,178 | 78.28 |
| ERR164593 | Adenocarcinoma | 39,817,881 | 35,177,832 | 88.35 | 27,640,638 | 78.57 |
| ERR164594 | Adenocarcinoma | 35,208,393 | 30,706,097 | 87.21 | 26,019,477 | 84.74 |
| ERR164595 | Adenocarcinoma | 55,195,368 | 42,244,155 | 76.54 | 34,149,671 | 80.84 |
| ERR164596 | Adenocarcinoma | 10,939,116 | 8,714,239 | 79.66 | 6,949,896 | 79.75 |
| ERR164598 | Adenocarcinoma | 16,098,438 | 12,856,904 | 79.86 | 10,243,579 | 79.67 |
| ERR164600 | Adenocarcinoma | 96,081,958 | 74,660,575 | 77.71 | 59,671,122 | 79.92 |
| ERR164601 | Adenocarcinoma | 60,414,304 | 47,203,775 | 78.13 | 36,247,327 | 76.79 |
| ERR164602 | Adenocarcinoma | 57,662,415 | 44,384,853 | 76.97 | 34,785,087 | 78.37 |
| ERR164603 | Adenocarcinoma | 63,856,760 | 48,920,665 | 76.61 | 36,730,661 | 75.08 |
| ERR164604 | Adenocarcinoma | 52,237,502 | 39,841,718 | 76.27 | 32,503,367 | 81.58 |
| ERR164605 | Adenocarcinoma | 55,302,491 | 41,749,397 | 75.49 | 31,391,333 | 75.19 |
| ERR164606 | Adenocarcinoma | 65,138,060 | 54,258,800 | 83.3 | 41,916,928 | 77.25 |
| ERR164607 | Adenocarcinoma | 61,863,976 | 51,017,834 | 82.47 | 39,874,456 | 78.16 |
| ERR164608 | Adenocarcinoma | 60,720,273 | 50,068,318 | 82.46 | 40,678,758 | 81.25 |
| ERR164609 | Adenocarcinoma | 57,606,955 | 46,709,175 | 81.08 | 36,005,051 | 77.08 |
| ERR164610 | Adenocarcinoma | 54,933,146 | 44,761,261 | 81.48 | 36,174,932 | 80.82 |
| ERR164611 | Adenocarcinoma | 63,317,174 | 51,919,415 | 82 | 40,131,899 | 77.3 |
| ERR164612 | Adenocarcinoma | 55,724,222 | 46,240,053 | 82.98 | 36,878,646 | 79.75 |
| ERR164613 | Adenocarcinoma | 67,129,037 | 55,415,448 | 82.55 | 44,169,645 | 79.71 |
| ERR164614 | Adenocarcinoma | 57,751,163 | 49,736,703 | 86.12 | 39,953,394 | 80.33 |
| ERR164615 | Adenocarcinoma | 63,332,457 | 54,832,993 | 86.58 | 45,428,012 | 82.85 |
| ERR164616 | Adenocarcinoma | 61,887,903 | 53,396,292 | 86.28 | 40,731,143 | 76.28 |
| ERR164617 | Adenocarcinoma | 54,159,676 | 46,427,255 | 85.72 | 36,509,256 | 78.64 |
| ERR164618 | Adenocarcinoma | 49,749,450 | 41,398,048 | 83.21 | 33,243,653 | 80.3 |
| ERR164619 | Adenocarcinoma | 67,385,676 | 55,488,112 | 82.34 | 44,647,294 | 80.46 |
| ERR164620 | Adenocarcinoma | 60,253,340 | 50,097,255 | 83.14 | 42,317,075 | 84.47 |
| ERR164621 | Adenocarcinoma | 66,588,273 | 55,095,900 | 82.74 | 46,408,061 | 84.23 |
| ERR164622 | Adenocarcinoma | 33,747,468 | 24,344,220 | 72.14 | 18,236,217 | 74.91 |
| ERR164623 | Adenocarcinoma | 34,223,354 | 24,667,623 | 72.08 | 19,702,829 | 79.87 |
| ERR164624 | Adenocarcinoma | 41,045,273 | 29,457,484 | 71.77 | 21,827,441 | 74.1 |
| ERR164625 | Adenocarcinoma | 34,893,898 | 25,374,988 | 72.72 | 19,222,559 | 75.75 |
| ERR164626 | Adenocarcinoma | 34,779,422 | 25,150,430 | 72.31 | 19,119,921 | 76.02 |
| ERR164627 | Adenocarcinoma | 37,334,315 | 27,377,896 | 73.33 | 20,201,544 | 73.79 |
| ERR164628 | Adenocarcinoma | 36,607,936 | 26,840,099 | 73.32 | 20,070,506 | 74.78 |
| ERR164629 | Adenocarcinoma | 32,000,887 | 23,438,676 | 73.24 | 17,858,734 | 76.19 |
| ERR164630 | Adenocarcinoma | 32,015,184 | 23,588,440 | 73.68 | 18,055,336 | 76.54 |
| ERR164631 | Adenocarcinoma | 35,692,599 | 26,387,839 | 73.93 | 20,554,601 | 77.89 |
| ERR164632 | Adenocarcinoma | 35,163,857 | 26,119,903 | 74.28 | 20,470,279 | 78.37 |
| ERR164633 | Adenocarcinoma | 38,627,258 | 28,309,482 | 73.29 | 23,201,425 | 81.96 |
| ERR164634 | Adenocarcinoma | 33,115,066 | 21,315,096 | 64.37 | 17,059,881 | 80.04 |
| ERR164500 | Normal | 49,956,366 | 41,851,998 | 83.78 | 36,355,020 | 86.87 |
| ERR164501 | Normal | 46,875,690 | 39,405,166 | 84.06 | 33,721,896 | 85.58 |
| ERR164502 | Normal | 50,624,081 | 42,637,305 | 84.22 | 35,254,617 | 82.68 |
| ERR164503 | Normal | 45,655,261 | 38,530,698 | 84.39 | 33,564,514 | 87.11 |
| ERR164504 | Normal | 45,700,930 | 38,970,483 | 85.27 | 31,965,341 | 82.02 |
| ERR164505 | Normal | 45,440,332 | 37,037,822 | 81.51 | 31,855,047 | 86.01 |
| ERR164506 | Normal | 41,766,400 | 34,447,242 | 82.48 | 28,406,539 | 82.46 |
| ERR164507 | Normal | 36,620,147 | 29,811,053 | 81.41 | 25,318,942 | 84.93 |
| ERR164508 | Normal | 36,929,327 | 30,390,271 | 82.29 | 25,960,473 | 85.42 |
| ERR164509 | Normal | 44,219,885 | 36,269,023 | 82.02 | 30,645,029 | 84.49 |
| ERR164510 | Normal | 31,899,940 | 26,080,218 | 81.76 | 21,704,617 | 83.22 |
| ERR164511 | Normal | 31,290,309 | 27,347,802 | 87.4 | 23,037,031 | 84.24 |
| ERR164512 | Normal | 39,920,425 | 35,108,852 | 87.95 | 29,618,668 | 84.36 |
| ERR164513 | Normal | 36,408,281 | 31,933,796 | 87.71 | 26,768,017 | 83.82 |
| ERR164514 | Normal | 35,726,514 | 31,674,116 | 88.66 | 24,806,606 | 78.32 |
| ERR164515 | Normal | 31,497,329 | 27,848,655 | 88.42 | 23,460,608 | 84.24 |
| ERR164516 | Normal | 34,642,008 | 30,665,741 | 88.52 | 25,198,744 | 82.17 |
| ERR164517 | Normal | 31,193,217 | 27,554,463 | 88.33 | 22,765,264 | 82.62 |
| ERR164518 | Normal | 36,693,314 | 32,693,089 | 89.1 | 26,556,455 | 81.23 |
| ERR164519 | Normal | 34,871,202 | 30,554,167 | 87.62 | 23,741,019 | 77.7 |
| ERR164520 | Normal | 35,109,105 | 30,903,055 | 88.02 | 25,763,397 | 83.37 |
| ERR164521 | Normal | 36,070,935 | 31,725,469 | 87.95 | 27,565,214 | 86.89 |
| ERR164522 | Normal | 37,385,715 | 33,213,950 | 88.84 | 28,015,439 | 84.35 |
| ERR164523 | Normal | 33,943,554 | 29,764,123 | 87.69 | 25,415,821 | 85.39 |
| ERR164524 | Normal | 29,128,935 | 25,610,088 | 87.92 | 21,477,580 | 83.86 |
| ERR164525 | Normal | 37,843,199 | 34,246,352 | 90.5 | 28,540,721 | 83.34 |
| ERR164526 | Normal | 31,527,488 | 28,073,588 | 89.04 | 22,746,539 | 81.02 |
| ERR164527 | Normal | 33,656,997 | 30,561,111 | 90.8 | 24,935,272 | 81.59 |
| ERR164528 | Normal | 38,815,656 | 35,063,942 | 90.33 | 29,854,727 | 85.14 |
| ERR164529 | Normal | 39,369,979 | 35,109,960 | 89.18 | 29,471,675 | 83.94 |
| ERR164530 | Normal | 28,568,100 | 25,163,824 | 88.08 | 19,343,741 | 76.87 |
| ERR164531 | Normal | 33,008,257 | 29,364,370 | 88.96 | 23,669,184 | 80.61 |
| ERR164532 | Normal | 34,867,292 | 31,167,070 | 89.39 | 26,304,428 | 84.4 |
| ERR164533 | Normal | 38,983,377 | 34,712,025 | 89.04 | 27,817,638 | 80.14 |
| ERR164534 | Normal | 35,282,174 | 31,407,008 | 89.02 | 25,260,737 | 80.43 |
| ERR164535 | Normal | 17,270,628 | 14,071,923 | 81.48 | 10,962,952 | 77.91 |
| ERR164536 | Normal | 38,797,818 | 34,478,036 | 88.87 | 27,428,376 | 79.55 |
| ERR164537 | Normal | 28,086,427 | 24,496,871 | 87.22 | 19,908,261 | 81.27 |
| ERR164538 | Normal | 35,274,948 | 30,782,467 | 87.26 | 25,734,547 | 83.6 |
| ERR164539 | Normal | 52,034,997 | 44,899,158 | 86.29 | 36,723,495 | 81.79 |
| ERR164540 | Normal | 47,760,113 | 41,039,429 | 85.93 | 34,353,331 | 83.71 |
| ERR164541 | Normal | 46,858,874 | 39,914,868 | 85.18 | 33,608,285 | 84.2 |
| ERR164542 | Normal | 50,435,154 | 43,409,062 | 86.07 | 35,916,168 | 82.74 |
| ERR164543 | Normal | 43,998,403 | 36,566,986 | 83.11 | 30,217,737 | 82.64 |
| ERR164544 | Normal | 45,519,810 | 37,931,956 | 83.33 | 33,055,753 | 87.14 |
| ERR164545 | Normal | 52,624,636 | 43,891,530 | 83.4 | 37,161,530 | 84.67 |
| ERR164546 | Normal | 38,877,419 | 31,842,890 | 81.91 | 26,173,354 | 82.2 |
| ERR164547 | Normal | 37,039,026 | 30,900,855 | 83.43 | 25,240,882 | 81.68 |
| ERR164548 | Normal | 39,530,504 | 33,405,413 | 84.51 | 28,123,833 | 84.19 |
| ERR164549 | Normal | 46,480,356 | 39,157,180 | 84.24 | 32,672,467 | 83.44 |
