# Supplementary material for: Automated identification of reference genes based on RNA-seq data
Source: Biomed Eng Online. 2017 Aug 18;16(Suppl 1):65. doi: 10.1186/s12938-017-0356-5 (PMC5568602; doi:10.1186/s12938-017-0356-5)
Supplement: Supplementary file 2 — Additional file 2. Best RGs in olive tree pistil according to Fig. 2a, ranked by CV. They were obtained for different stages of pistil development with CV < 10% and minimum counted reads of 100. Transcript_id: transcript identifiers in the ReprOlive transcriptome. [file 12938_2017_356_MOESM2_ESM.docx]

**Additional File 2: Best RGs in olive tree pistil according to Figure 2A, ranked by CV.** They were obtained for different stages of pistil development with CV < 10% and minimum counted reads of 100. *Transcript_id*: transcript identifiers in the ReprOlive transcriptome.

| **PISTIL** | **RPMM** | | | **CV(%)** | **Mean RPMM** | **Best hit** | **Description** |
| --- | --- | --- | --- | --- | --- | --- | --- |
| **transcript_id** | **S2** | **S3** | **S4** |  |  |  |  |
| rp11_olive_000229 | 1,061 | 1,091 | 1,050 | 1.62 | 1,067.3 | A0A022R633 | Uncharacterized protein *Erythranthe guttata* |
| rp11_olive_006479 | 1,352 | 1,415 | 1,331 | 2.61 | 1,366 | P69310 | Ubiquitin *Avena sativa* |
| rp11_olive_019507 | 8,425 | 8,943 | 9,152 | 3.46 | 8,840 | Q6RYA0 | Salicylic acid-binding protein 2 *Nicotiana tabacum* |
| rp11_olive_015883 | 8,425 | 8,962 | 9,152 | 3.48 | 8,846.3 | Q8S8S9 | Methylesterase 1 *Arabidopsis thaliana* |
| rp11_olive_018099 | 1,727 | 1,779 | 1,892 | 3.83 | 1,799.3 | Q39196 | Probable aquaporin PIP1-4 *Arabidopsis thaliana* |
| rp11_olive_006473 | 2,704 | 2,673 | 2,408 | 5.12 | 2,595 | Q8H159 | Polyubiquitin 10 *Arabidopsis thaliana* |
| rp11_olive_031243 | 1,768 | 1,533 | 1,702 | 5.93 | 1,667.7 | P69313 | Ubiquitin *Helianthus annuus* |
| rp11_olive_045557 | 1,113 | 1,032 | 960 | 6.04 | 1,035 | K7US22 | Ubiquitin2 *Zea mays* |
| rp11_olive_019545 | 4,587 | 5,218 | 5,467 | 7.28 | 5,090.7 | D7TBK4 | Putative uncharacterized protein *Vitis vinifera* |
| rp11_olive_008243 | 1,810 | 1,543 | 1,829 | 7.56 | 1,727.3 | O64937 | Elongation factor 1-alpha *Oryza sativa* |
| rp11_olive_003751 | 1,487 | 1,366 | 1,222 | 7.97 | 1,358.3 | P26520 | Glyceraldehyde-3-phosphate dehydrogenase cytosolic *Petunia hybrida* |
| rp11_olive_000305 | 1,217 | 1,061 | 987 | 8.81 | 1,088.3 | Q84JM4 | Topless-related protein 3 *Arabidopsis thaliana* |
| rp11_olive_004563 | 1,477 | 1,749 | 1,847 | 9.26 | 1,691 | Q96471 | S-adenosylmethionine decarboxylase proenzyme *Ipomoea nil* |
| rp11_olive_002595 | 1,206 | 1,081 | 960 | 9.28 | 1,082.3 | O24661 | Asparagine synthetase *Triphysaria versicolor* |
| rp11_olive_009319 | 1,883 | 1,582 | 2,000 | 9.67 | 1,821.7 | P17786 | Elongation factor 1-alpha *Solanum lycopersicum* |
