# Supplementary material for: Automated identification of reference genes based on RNA-seq data
Source: Biomed Eng Online. 2017 Aug 18;16(Suppl 1):65. doi: 10.1186/s12938-017-0356-5 (PMC5568602; doi:10.1186/s12938-017-0356-5)
Supplement: Supplementary file 3 — Additional file 3. Best RGs in olive tree pollen according to Fig. 2b, ranked by CV. They were obtained for different stages of pollen development, with CV < 10% and minimum counted reads of 100. Transcript_id: transcript identifiers in the ReprOlive transcriptome. [file 12938_2017_356_MOESM3_ESM.docx]

**Additional File 3: Best RGs in olive tree pollen according to Figure 2B, ranked by CV.** They were obtained for different stages of pollen development, with CV < 10% and minimum counted reads of 100. *Transcript_id*: transcript identifiers in the ReprOlive transcriptome

| **POLLEN** | **RPMM** | | | **CV(%)** | **Mean RPMM** | **Best hit** | **Description** |
| --- | --- | --- | --- | --- | --- | --- | --- |
| **transcript_id** | **PM** | **PG1** | **PG5** |  |  |  |  |
| rp11_olive_000239 | 1,021 | 992 | 996 | 1.28 | 1,003 | Q8GYX0 | MOB kinase activator-like 1 *Arabidopsis* *thaliana* |
| rp11_olive_006957 | 910 | 877 | 884 | 1.59 | 890.3 | Q2QYH7 | Cytochrome P450 714C2 *Oryza* *sativa* |
| rp11_olive_003317 | 755 | 775 | 740 | 1.89 | 756.7 | Q9LWM4 | CBL-interacting protein kinase 5 *Oryza* *sativa* |
| rp11_olive_006483_split_1 | 1,365 | 1,311 | 1,373 | 2.04 | 1,349.7 | D9ZHD7 | Polygalacturonase *Hypericum* *perforatum* |
| rp11_olive_002039 | 999 | 1,017 | 1,054 | 2.24 | 1,023.3 | O23492 | Inositol transporter 4 *Arabidopsis* *thaliana* |
| rp11_olive_008165 | 838 | 800 | 776 | 3.17 | 804.7 | P48631 | Omega-6 fatty acid desaturase, endoplasmic reticulum isozyme 2 *Glycine* *max* |
| rp11_olive_003081 | 1,948 | 2,111 | 2,037 | 3.28 | 2,032 | Q94A34 | Phosphatidylinositol/phosphatidylcholine transfer protein SFH12 *Arabidopsis* *thaliana* |
| rp11_olive_003685 | 1,038 | 975 | 956 | 3.54 | 989.7 | A0A022QFM6 | Uncharacterized protein *Erythranthe* *guttata* |
| rp11_olive_007869 | 2,824 | 3,052 | 2,836 | 3.61 | 2,904 | Q9LSE7 | Cytochrome b561 and DOMON domain-containing protein At3g25290 *Arabidopsis* *thaliana* |
| rp11_olive_002783 | 771 | 834 | 772 | 3.72 | 792.3 | C0LGU0 | Probable LRR receptor-like serine/threonine-protein kinase RLK *Arabidopsis* *thaliana* |
| rp11_olive_001165_split_0 | 8,063 | 8,850 | 8,333 | 3.88 | 8,415.3 | Q9SBA7 | Sugar transport protein 8 *Arabidopsis* *thaliana* |
| rp11_olive_005581 | 1,204 | 1,315 | 1,221 | 3.92 | 1,246.7 | Q96471 | S-adenosylmethionine decarboxylase proenzyme *Ipomoea* *nil* |
| rp11_olive_004159 | 6,648 | 7,275 | 6,740 | 4.01 | 6,887.7 | Q43043 | Pectinesterase *Petunia* *integrifolia* |
| rp11_olive_007853 | 2,924 | 3,231 | 3,078 | 4.07 | 3,077.7 | B2VPR8 | Pectinesterase 2 *Olea* *europaea* |
| rp11_olive_023981 | 1,476 | 1,630 | 1,602 | 4.27 | 1,569.3 | P27164 | Calmodulin-related protein *Petunia* *hybrida* |
| rp11_olive_005653 | 1,093 | 1,043 | 1,158 | 4.29 | 1,098 | P43297 | Cysteine proteinase RD21a *Arabidopsis* *thaliana* |
| rp11_olive_006745 | 621 | 596 | 556 | 4.53 | 591 | Q84KJ6 | Ammonium transporter 3 member 1 *Oryza* *sativa* |
| rp11_olive_013493 | 949 | 954 | 1,046 | 4.54 | 983 | A0A022Q3I8 | Actin-7 *Arabidopsis* *thaliana* |
| rp11_olive_015265 | 1,382 | 1,528 | 1,539 | 4.83 | 1,483 | O23609 | Peroxidase 41 *Arabidopsis* *thaliana* |
| rp11_olive_001387 | 799 | 860 | 763 | 4.96 | 807.3 | Q9SIT5 | Cation/H(+) antiporter 15 *Arabidopsis* *thaliana* |
| rp11_olive_030809 | 927 | 851 | 960 | 5 | 912.7 | Q05349 | Auxin-repressed 12.5 kDa protein *Fragaria* *ananassa* |
| rp11_olive_001361 | 816 | 898 | 803 | 5.01 | 839 | A0A068U7U3 | Coffea canephora DH200=94 genomic scaffold, scaffold_12 *Coffea* *canephora* |
| rp11_olive_007883 | 860 | 758 | 830 | 5.25 | 816 | A0A022QM75 | Uncharacterized protein *Erythranthe* *guttata* |
| rp11_olive_007927 | 4,389 | 4,891 | 4,375 | 5.27 | 4,551.7 | A5BLS9 | Putative uncharacterized protein *Vitis* *vinifera* |
| rp11_olive_007827 | 1,054 | 924 | 978 | 5.41 | 985.3 | A0A068TST0 | Coffea canephora DH200=94 genomic scaffold, scaffold_3 *Coffea* *canephora* |
| rp11_olive_010119 | 560 | 583 | 637 | 5.44 | 593.3 | Q9ZT42 | E3 ubiquitin-protein ligase RHF2A *Arabidopsis* *thaliana* |
| rp11_olive_000843_split_1 | 2,430 | 2,669 | 2,778 | 5.54 | 2,625.7 | M1AUV5 | Uncharacterized protein *Solanum* *tuberosum* |
| rp11_olive_069439 | 2,380 | 2,422 | 2,697 | 5.62 | 2,499.7 | Q6NMJ2 | Pollen-specific protein-like At4g18596 *Arabidopsis* *thaliana* |
| rp11_olive_007141 | 566 | 575 | 642 | 5.7 | 594.3 | B9N2R5 | Hydroxyproline-rich glycoprotein *Populus* *trichocarpa* |
| rp11_olive_006487 | 982 | 885 | 853 | 6.05 | 906.7 | P92961 | Proline transporter 1 *Arabidopsis* *thaliana* |
| rp11_olive_006915 | 7,053 | 8,135 | 7,215 | 6.38 | 7,467.7 | P15722 | Probable pectate lyase P59 *Solanum* *lycopersicum* |
| rp11_olive_011687 | 10,277 | 10,596 | 11,909 | 6.46 | 10,927.3 | Q9LK32 | Uncharacterized protein At3g27210 *Arabidopsis* *thaliana* |
| rp11_olive_008617 | 1,254 | 1,473 | 1,337 | 6.66 | 1,354.7 | Q94G86 | Glucan endo-1,3-beta-D-glucosidase *Olea* *europaea* |
| rp11_olive_006113 | 572 | 498 | 579 | 6.67 | 549.7 | A0A068UGI7 | Coffea canephora DH200=94 genomic scaffold, scaffold_26 *Coffea* *canephora* |
| rp11_olive_008709 | 2,003 | 2,133 | 1,799 | 6.95 | 1,978.3 | Q766C3 | Aspartic proteinase nepenthesin-1 *Nepenthes* *gracilis* |
| rp11_olive_012135 | 666 | 626 | 561 | 7.01 | 617.7 | Q8VZD5 | Shaggy-related protein kinase epsilon *Arabidopsis* *thaliana* |
| rp11_olive_005053 | 5,960 | 6,104 | 5,178 | 7.08 | 5,747.3 | Q05967 | Polygalacturonase *Nicotiana* *tabacum* |
| rp11_olive_001243 | 2,958 | 3,001 | 2,553 | 7.11 | 2,837.3 | Q9SCU9 | Beta-galactosidase 13 *Arabidopsis* *thaliana* |
| rp11_olive_001611 | 921 | 966 | 808 | 7.4 | 898.3 | A0A022QCS3 | Uncharacterized protein *Erythranthe* *guttata* |
| rp11_olive_002553 | 4,223 | 4,729 | 5,066 | 7.41 | 4,672.7 | Q8VYG3 | Endoglucanase 16 *Arabidopsis* *thaliana* |
| rp11_olive_003175 | 2,247 | 2,652 | 2,302 | 7.47 | 2,400.3 | P43390 | Metallothionein-like protein type 2 *Actinidia* *deliciosa* |
| rp11_olive_006165 | 993 | 1,141 | 965 | 7.48 | 1,033 | A0A068U288 | Coffea canephora DH200=94 genomic scaffold, scaffold_8 *Coffea* *canephora* |
| rp11_olive_016425_split_1 | 3,840 | 4,606 | 4,124 | 7.55 | 4,190 | Q9ZQ23 | Fasciclin-like arabinogalactan protein 3 *Arabidopsis* *thaliana* |
| rp11_olive_004881 | 14,105 | 15,236 | 12,649 | 7.57 | 13,996.7 | A0A022S280 | Pectinesterase *Erythranthe* *guttata* |
| rp11_olive_002483 | 1,648 | 1,639 | 1,391 | 7.64 | 1,559.3 | Q9LZR3 | Glucomannan 4-beta-mannosyltransferase 9 *Arabidopsis* *thaliana* |
| rp11_olive_019347 | 11,298 | 12,039 | 13,574 | 7.7 | 12,303.7 | Q6U740 | Allergen Fra e 1 *Fraxinus* *excelsior* |
| rp11_olive_002709 | 2,236 | 2,146 | 1,858 | 7.75 | 2,080 | Q9FE20 | Serine/threonine-protein kinase PBS1 *Arabidopsis* *thaliana* |
| rp11_olive_009557 | 1,199 | 1,043 | 1,005 | 7.76 | 1,082.3 | Q9SJP9 | Proline transporter 3 *Arabidopsis* *thaliana* |
| rp11_olive_009589 | 1,099 | 932 | 933 | 7.94 | 988 | P92962 | Proline transporter 2 *Arabidopsis* *thaliana* |
| rp11_olive_000913 | 1,665 | 1,771 | 1,458 | 7.97 | 1,631.3 | A0A068V323 | Coffea canephora DH200=94 genomic scaffold, scaffold_92 *Coffea* *canephora* |
| rp11_olive_011311 | 971 | 843 | 808 | 8.02 | 874 | P92962-2 | 2 of Proline transporter 2 *Arabidopsis* *thaliana* |
| rp11_olive_005955 | 2,486 | 2,652 | 3,011 | 8.07 | 2,716.3 | Q4PNY1 | Expansin-A11 *Oryza* *sativa* |
| rp11_olive_001657 | 688 | 617 | 565 | 8.09 | 623.3 | Q9MAP5 | Subtilisin-like protease SBT3.3 *Arabidopsis* *thaliana* |
| rp11_olive_014633 | 732 | 877 | 754 | 8.1 | 787.7 | M1A3A5 | Uncharacterized protein *Solanum* *tuberosum* |
| rp11_olive_021833 | 4,594 | 4,989 | 5,595 | 8.14 | 5,059.3 | Q05431 | L-ascorbate peroxidase 1, cytosolic *Arabidopsis* *thaliana* |
| rp11_olive_026419 | 2,308 | 2,473 | 2,804 | 8.16 | 2,528.3 | Q6YYW5 | Expansin-A32 *Oryza* *sativa* |
| rp11_olive_004701 | 710 | 660 | 579 | 8.31 | 649.7 | O04057 | Aspartic proteinase *Cucurbita* *pepo* |
| rp11_olive_002301 | 9,672 | 11,021 | 9,028 | 8.38 | 9,907 | O22149 | Probable pectinesterase/pectinesterase inhibitor 17 *Arabidopsis* *thaliana* |
| rp11_olive_009101 | 3,468 | 4,265 | 3,819 | 8.47 | 3,850.7 | D8VPP5 | Pectinesterase 1 *Olea* *europaea* |
| rp11_olive_021197 | 2,097 | 2,265 | 2,571 | 8.49 | 2,311 | Q9M2S9 | Expansin-A16 *Arabidopsis* *thaliana* |
| rp11_olive_005937 | 1,365 | 1,337 | 1,122 | 8.52 | 1,274.7 | Q96287 | Shaggy-related protein kinase theta *Arabidopsis* *thaliana* |
| rp11_olive_003945 | 2,036 | 1,958 | 1,660 | 8.6 | 1,884.7 | P53492 | Actin-7 *Arabidopsis* *thaliana* |
| rp11_olive_071538 | 3,518 | 3,857 | 4,339 | 8.63 | 3,904.7 | Q9M373 | Arabinogalactan peptide 20 *Arabidopsis* *thaliana* |
| rp11_olive_003559 | 755 | 805 | 651 | 8.7 | 737 | Q8W034 | Heterogeneous nuclear ribonucleoprotein 1 *Arabidopsis* *thaliana* |
| rp11_olive_003491 | 771 | 847 | 682 | 8.8 | 766.7 | Q94CB1-2 | 2 of Probable pectinesterase/pectinesterase inhibitor 25 *Arabidopsis* *thaliana* |
| rp11_olive_025593 | 677 | 822 | 821 | 8.81 | 773.3 | A0A022QLI1 | Uncharacterized protein *Erythranthe* *guttata* |
| rp11_olive_005301 | 1,448 | 1,775 | 1,737 | 8.83 | 1,653.3 | P26792 | Beta-fructofuranosidase, insoluble isoenzyme 1 *Daucus* *carota* |
| rp11_olive_010915 | 1,243 | 1,090 | 1,005 | 8.85 | 1,112.7 | W9R1P4 | Lysine histidine transporter 1 *Morus* *notabilis*. |
| rp11_olive_002287 | 1,187 | 1,073 | 951 | 9 | 1,070.3 | M1BB40 | Uncharacterized protein *Solanum* *tuberosum* |
| rp11_olive_011807 | 1,043 | 1,043 | 1,261 | 9.21 | 1,115.7 | A0A061EN15 | Adenine nucleotide alpha hydrolases-like superfamily protein, putative *Theobroma* *cacao* |
| rp11_olive_026709 | 810 | 890 | 1,014 | 9.28 | 904.7 | Q9MA62 | Protein RALF-like 22 *Arabidopsis* *thaliana* |
| rp11_olive_003803 | 1,864 | 1,754 | 1,485 | 9.36 | 1,701 | Q9ZPY9 | Phosphatidylinositol 4-kinase gamma 4 *Arabidopsis* *thaliana* |
| rp11_olive_034631 | 1,354 | 1,477 | 1,696 | 9.37 | 1,509 | R0G2X7 | Uncharacterized protein *Capsella* *rubella*. |
| rp11_olive_003735 | 744 | 826 | 655 | 9.42 | 741.7 | Q3E954 | Probable boron transporter 6 *Arabidopsis* *thaliana* |
| rp11_olive_070164 | 1,709 | 2,005 | 2,154 | 9.46 | 1,956 | P13447 | Anther-specific protein LAT52 *Solanum* *lycopersicum* |
| rp11_olive_000235_split_1 | 8,362 | 8,923 | 7,063 | 9.6 | 8,116 | Q9M2A0 | ATPase 8, plasma membrane-type *Arabidopsis* *thaliana* |
| rp11_olive_000981 | 721 | 805 | 633 | 9.76 | 719.7 | Q9FMR9 | RuvB-like protein 1 *Arabidopsis* *thaliana* |
