# Supplementary material for: Automated identification of reference genes based on RNA-seq data
Source: Biomed Eng Online. 2017 Aug 18;16(Suppl 1):65. doi: 10.1186/s12938-017-0356-5 (PMC5568602; doi:10.1186/s12938-017-0356-5)
Supplement: Supplementary file 4 — Additional file 4. Best candidate RGs in Arabidopsis thaliana for replicates 1, 2 and 3 with their respective RPMM values for each replicate. They were obtained with CV < 10% and a minimum counted reads of 10,000. Transcript_id: transcript identifiers in TAIR database. [file 12938_2017_356_MOESM4_ESM.docx]

**Additional File 4: Best candidate RGs in *Arabidopsis thaliana* for replicates 1, 2 and 3 with their respective RPMM values for each replicate.** They were obtained with CV < 10% and a minimum counted reads of 10,000. *Transcript_id*: transcript identifiers in TAIR database.

| transcript_id | Description | REPLICATE 1 | | REPLICATE 2 | | REPLICATE 3 | |
| --- | --- | --- | --- | --- | --- | --- | --- |
|  |  | CV(%) | Mean RPMM | CV(%) | Mean RPMM | CV(%) | Mean RPMM |
| AT1G59870 | ABC-2 and Plant PDR ABC-type transporter family protein | 8.99 | 445 | 6.16 | 462.5 | 9.57 | 454.5 |
| AT3G08580 | ADP/ATP carrier 1 | 4.85 | 669.5 | 2.01 | 670.5 | 1.38 | 688.5 |
| AT2G13360 | alanine:glyoxylate aminotransferase | 6.08 | 576 | 1.35 | 557.5 | 3.95 | 569.5 |
| AT3G52930 | Aldolase superfamily protein | 8.21 | 487 | 1.23 | 487 | 1.6 | 500 |
| AT3G14415 | Aldolase-type TIM barrel family protein | 5.05 | 822.5 | 5.67 | 793 | 4.52 | 829.5 |
| AT3G14420 | Aldolase-type TIM barrel family protein | 3.05 | 952 | 4.32 | 913.5 | 4.23 | 969 |
| AT5G08670 | ATP synthase alpha/beta family protein | 2.17 | 438.5 | 6.32 | 443 | 6.54 | 435.5 |
| AT5G08680 | ATP synthase alpha/beta family protein | 3.91 | 384 | 8.71 | 379 | 7.37 | 380 |
| AT5G08690 | ATP synthase alpha/beta family protein | 3.34 | 463.5 | 7.53 | 465 | 6.72 | 461 |
| AT4G32260 | ATPase, F0 complex, subunit B/B', bacterial/chloroplast | 5.03 | 477 | 2.11 | 450.5 | 5.03 | 477 |
| AT4G04640 | ATPase, F1 complex, gamma subunit protein | 1.27 | 550 | 0 | 511 | 2.29 | 546.5 |
| AT3G01500 | carbonic anhydrase 1 | 0.8 | 1617 | 6.65 | 1519 | 0.54 | 1768.5 |
| AT5G14740 | carbonic anhydrase 2 | 2.3 | 566 | 5.84 | 548 | 0.49 | 618 |
| AT1G09340 | chloroplast RNA binding | 7.78 | 450 | 0.81 | 429.5 | 6.11 | 491 |
| AT5G17920 | Cobalamin-independent synthase family protein | 4.24 | 1251 | 1.53 | 1239 | 2.09 | 1245 |
| AT3G02468 | conserved peptide upstream open reading frame 9 | 8.44 | 794 | 7.39 | 798 | 5.96 | 796.5 |
| AT1G54410 | dehydrin family protein | 3.23 | 433 | 4.55 | 450.5 | 3.07 | 439.5 |
| AT2G36530 | Enolase | 3.83 | 574 | 0.26 | 578.5 | 2 | 575.5 |
| AT4G25100 | Fe superoxide dismutase 1 | 1.2 | 543.5 | 2.32 | 517 | 2.53 | 514 |
| AT5G66190 | ferredoxin-NADP(+)-oxidoreductase 1 | 1.06 | 472 | 2.77 | 451.5 | 0.99 | 503 |
| AT2G21330 | fructose-bisphosphate aldolase 1 | 6.61 | 635 | 0.16 | 624 | 8.15 | 687 |
| AT4G38970 | fructose-bisphosphate aldolase 2 | 0.83 | 1387.5 | 3.75 | 1345.5 | 2.12 | 1462 |
| AT5G46110 | Glucose-6-phosphate/phosphate translocator-related | 0.76 | 595.5 | 1.3 | 577.5 | 4.09 | 611 |
| AT5G04140 | glutamate synthase 1 | 1.18 | 847 | 4.62 | 822 | 2.25 | 865.5 |
| AT1G23310 | glutamate:glyoxylate aminotransferase | 2.26 | 908.5 | 2.14 | 865.5 | 4.36 | 894 |
| AT5G35630 | glutamine synthetase 2 | 4.31 | 892.5 | 0.46 | 862 | 5.82 | 936.5 |
| AT3G26650 | glyceraldehyde 3-phosphate dehydrogenase A subunit | 0.26 | 1137 | 2.37 | 1075.5 | 2.45 | 1161.5 |
| AT1G42970 | glyceraldehyde-3-phosphate dehydrogenase B subunit | 4.27 | 1031 | 7.04 | 1008 | 0.86 | 1109.5 |
| AT3G04120 | glyceraldehyde-3-phosphate dehydrogenase C subunit 1 | 1.15 | 694 | 4.9 | 704.5 | 8.5 | 718 |
| AT1G13440 | glyceraldehyde-3-phosphate dehydrogenase C2 | 4.08 | 932 | 0.39 | 898.5 | 2.53 | 947 |
| AT1G11860 | Glycine cleavage T-protein family | 2.09 | 621 | 4.88 | 583.5 | 3.98 | 665.5 |
| AT4G33010 | glycine decarboxylase P-protein 1 | 4.81 | 1184 | 2.66 | 1164 | 4.18 | 1184.5 |
| AT2G26080 | glycine decarboxylase P-protein 2 | 5.07 | 522.5 | 3.94 | 508 | 7.48 | 508 |
| AT1G07920 | GTP binding Elongation factor Tu family protein | 1.76 | 1365 | 9.79 | 1348 | 0.7 | 1351.5 |
| AT1G07930 | GTP binding Elongation factor Tu family protein | 1.35 | 1331 | 9.56 | 1312.5 | 1.26 | 1313.5 |
| AT1G07940 | GTP binding Elongation factor Tu family protein | 1.66 | 1355.5 | 9.6 | 1334 | 0.52 | 1336 |
| AT5G60390 | GTP binding Elongation factor Tu family protein | 1.56 | 1414 | 8.41 | 1403 | 3.72 | 1409.5 |
| AT2G18960 | H(+)-ATPase 1 | 8.44 | 545 | 4.77 | 545 | 8.46 | 520 |
| AT4G30190 | H(+)-ATPase 2 | 1.19 | 545.5 | 4.94 | 587 | 7.27 | 578 |
| AT5G02500 | heat shock cognate protein 70-1 | 0.5 | 901.5 | 1.87 | 908 | 0.24 | 837 |
| AT5G28540 | heat shock protein 70 (Hsp 70) family protein | 4.08 | 428.5 | 6.32 | 419.5 | 8.28 | 428.5 |
| AT5G42020 | Heat shock protein 70 (Hsp 70) family protein | 3.94 | 431 | 5.8 | 422.5 | 7.93 | 429 |
| AT5G56030 | heat shock protein 81-2 | 1.22 | 490 | 7.18 | 480.5 | 2.94 | 458.5 |
| AT5G56010 | heat shock protein 81-3 | 1.31 | 494.5 | 7 | 486 | 2.81 | 462 |
| AT1G15690 | Inorganic H pyrophosphatase family protein | 9.16 | 546 | 7.52 | 538.5 | 8.63 | 527.5 |
| AT1G04410 | Lactate/malate dehydrogenase family protein | 0.61 | 495 | 1.9 | 499.5 | 2.3 | 500.5 |
| AT5G02380 | metallothionein 2B | 3.7 | 568 | 1.27 | 553 | 1.72 | 495.5 |
| AT5G53460 | NADH-dependent glutamate synthase 1 | 0.31 | 478.5 | 3.36 | 506 | 2.22 | 495 |
| AT1G37130 | nitrate reductase 2 | 4.81 | 520 | 8 | 525 | 3 | 517.5 |
| AT4G21960 | Peroxidase superfamily protein | 3.02 | 1077.5 | 8.25 | 1079 | 7.34 | 973.5 |
| AT5G09660 | peroxisomal NAD-malate dehydrogenase 2 | 3.15 | 460.5 | 0.89 | 448 | 4.49 | 456.5 |
| AT3G12780 | phosphoglycerate kinase 1 | 2.57 | 759.5 | 0.69 | 720 | 5.1 | 774.5 |
| AT1G32060 | phosphoribulokinase | 3.15 | 746.5 | 1.54 | 716 | 2.67 | 748 |
| AT4G03280 | photosynthetic electron transfer C | 6.44 | 559 | 5.1 | 529 | 6.49 | 577.5 |
| AT4G21280 | photosystem II subunit QA | 5.57 | 664 | 0.47 | 633 | 7.18 | 703.5 |
| AT2G06520 | photosystem II subunit X | 5.48 | 712 | 7.59 | 685 | 8.74 | 732 |
| AT2G45960 | plasma membrane intrinsic protein 1B | 0.45 | 448 | 3.64 | 439 | 2.8 | 429 |
| AT4G20360 | RAB GTPase homolog E1B | 0.34 | 597 | 6.23 | 594 | 4.69 | 618 |
| AT1G43170 | ribosomal protein 1 | 2.95 | 440 | 8.63 | 452 | 1.69 | 444.5 |
| AT1G02780 | Ribosomal protein L19e family protein | 1.8 | 444 | 6.36 | 440 | 1.9 | 446.5 |
| AT3G25520 | ribosomal protein L5 | 4.53 | 508 | 7.19 | 493.5 | 5.18 | 502 |
| AT5G39740 | ribosomal protein L5 B | 4.53 | 452.5 | 6.94 | 439.5 | 5.25 | 447.5 |
| AT1G56070 | Ribosomal protein S5/Elongation factor G/III/V family protein | 1.04 | 820.5 | 5.58 | 806 | 1.07 | 793.5 |
| AT5G20290 | Ribosomal protein S8e family protein | 2.98 | 436 | 9.3 | 435.5 | 4.7 | 436.5 |
| AT5G38410 | Ribulose bisphosphate carboxylase (small chain) family protein | 2.36 | 7947.5 | 8.98 | 7742.5 | 1.95 | 8997 |
| AT1G67090 | ribulose bisphosphate carboxylase small chain 1A | 1.53 | 9333.5 | 6.13 | 9079.5 | 6.54 | 10372.5 |
| AT2G39730 | rubisco activase | 3.48 | 4197 | 5.07 | 4114.5 | 1.79 | 4444.5 |
| AT4G13940 | S-adenosyl-L-homocysteine hydrolase | 2.65 | 792 | 6.43 | 785.5 | 6.37 | 777.5 |
| AT3G02470 | S-adenosylmethionine decarboxylase | 8.45 | 793 | 7.4 | 797 | 5.97 | 795.5 |
| AT3G17390 | S-adenosylmethionine synthetase family protein | 0.35 | 424.5 | 5.03 | 427.5 | 3.78 | 397 |
| AT3G55800 | sedoheptulose-bisphosphatase | 4.98 | 552.5 | 0.19 | 514 | 6.68 | 569 |
| AT4G37930 | serine transhydroxymethyltransferase 1 | 0.56 | 1166.5 | 1.62 | 1112 | 0.6 | 1165 |
| AT3G60750 | Transketolase | 7.77 | 1222 | 6.2 | 1146 | 3.47 | 1195.5 |
| AT3G55440 | triosephosphate isomerase | 0.97 | 413 | 0.98 | 409 | 7.62 | 407 |
| AT1G50010 | tubulin alpha-2 chain | 1.67 | 746.5 | 6.93 | 728.5 | 0.99 | 705 |
| AT1G04820 | tubulin alpha-4 chain | 2.75 | 745.5 | 8.19 | 726.5 | 0 | 701 |
| AT4G14960 | Tubulin/FtsZ family protein | 1.98 | 756 | 7.13 | 736.5 | 0.7 | 710 |
