# Supplementary material for: Automated identification of reference genes based on RNA-seq data
Source: Biomed Eng Online. 2017 Aug 18;16(Suppl 1):65. doi: 10.1186/s12938-017-0356-5 (PMC5568602; doi:10.1186/s12938-017-0356-5)
Supplement: Supplementary file 5 — Additional file 5. Best candidate RGs for normal lung tissues according to Fig. 6a and ranked by CV. They were obtained with CV < 15% and minimum counted reads of 10,000. Transcript_id: human transcript identifiers in ENSEMBL database. [file 12938_2017_356_MOESM5_ESM.docx]

**Additional File 5: Best candidate RGs for normal lung tissues according to Figure 6A and ranked by CV.** They were obtained with CV < 15% and minimum counted reads of 10,000. *Transcript_id*: human transcript identifiers in ENSEMBL database.

| **Transcript_id** | **CV(%)** | **Mean RPMM** | **Gene** | **Description** |
| --- | --- | --- | --- | --- |
| ENST00000338970.10 | 10.5 | 109 | RPL14 | ribosomal protein L14 |
| ENST00000412331.6 | 10.84 | 63.6 | EIF3L | eukaryotic translation initiation factor 3 subunit L |
| ENST00000401722.7 | 10.88 | 76 | SLC25A3 | solute carrier family 25 (mitochondrial carrier; phosphate carrier), member 3 |
| ENST00000425566.1 | 11.28 | 73.1 | RPL23AP87 | ribosomal protein L23a pseudogene 87 |
| ENST00000353411.10 | 11.64 | 60.3 | SKP1 | S-phase kinase-associated protein 1 |
| ENST00000510199.5 | 12.26 | 75.3 | GNB2L1 | guanine nucleotide binding protein (G protein), beta polypeptide 2-like 1 |
| ENST00000356769.7 | 12.28 | 65.9 | NACA | nascent polypeptide-associated complex alpha subunit |
| ENST00000261890.6 | 12.29 | 58.1 | RAB11A | RAB11A, member RAS oncogene family |
| ENST00000438331.5 | 13.13 | 52 | ANXA11 | annexin A11 |
| ENST00000586054.2 | 13.16 | 48.7 | OAZ1 | ornithine decarboxylase antizyme 1 |
| ENST00000456530.6 | 13.43 | 69.4 | RPL15 | ribosomal protein L15 |
| ENST00000420826.6 | 13.49 | 50 | SUMO2 | small ubiquitin-like modifier 2 |
| ENST00000426371.2 | 13.55 | 95.6 | HNRNPA1P40 | heterogeneous nuclear ribonucleoprotein A1 pseudogene 40 |
| ENST00000342374.4 | 13.57 | 58.3 | SERINC3 | serine incorporator 3 |
| ENST00000556083.1 | 13.91 | 71.8 | ACTN1 | actinin, alpha 1 |
| ENST00000360299.9 | 14.04 | 51.6 | RAB5B | RAB5B, member RAS oncogene family |
| ENST00000564521.6 | 14.08 | 86.5 | ALDOA | aldolase, fructose-bisphosphate A |
| ENST00000249822.8 | 14.18 | 66.9 | ARPP19 | cAMP regulated phosphoprotein 19kDa |
| ENST00000398752.10 | 14.18 | 101.9 | ATP5A1 | ATP synthase, H+ transporting, mitochondrial F1 complex, alpha subunit 1, cardiac muscle |
| ENST00000547276.5 | 14.3 | 65.9 | HNRNPA1 | heterogeneous nuclear ribonucleoprotein A1 |
| ENST00000261733.6 | 14.52 | 193.1 | ALDH2 | aldehyde dehydrogenase 2 family (mitochondrial) |
| ENST00000312239.9 | 14.69 | 48.7 | HP1BP3 | heterochromatin protein 1, binding protein 3 |
| ENST00000371065.8 | 14.75 | 70.9 | LEPROT | leptin receptor overlapping transcript |
| ENST00000571735.2 | 14.84 | 45.5 | BSG | basigin (Ok blood group) |
| ENST00000334660.9 | 14.85 | 89.1 | CHP1 | calcineurin-like EF-hand protein 1 |
| ENST00000500728.2 | 14.87 | 73.9 | LRPAP1 | LDL receptor related protein associated protein 1 |
| ENST00000334256.8 | 14.88 | 45.6 | KPNA4 | karyopherin alpha 4 (importin alpha 3) |
