# Supplementary material for: Automated identification of reference genes based on RNA-seq data
Source: Biomed Eng Online. 2017 Aug 18;16(Suppl 1):65. doi: 10.1186/s12938-017-0356-5 (PMC5568602; doi:10.1186/s12938-017-0356-5)
Supplement: Supplementary file 6 — Additional file 6. Best candidate RGs for normal and malignant lung samples according to Fig. 6b, ranked by CV. They were obtained with CV < 20% and minimum counted reads of 10,000. Transcript_id: human transcript identifiers in ENSEMBL database. [file 12938_2017_356_MOESM6_ESM.docx]

**Additional File 6: Best candidate RGs for normal and malignant lung samples according to Figure 6B, ranked by CV.** They were obtained with CV < 20% and minimum counted reads of 10,000. *Transcript_id*: human transcript identifiers in ENSEMBL database.

| **Transcript_id** | **CV(%)** | **Mean RPMM** | **Gene** | **Description** |
| --- | --- | --- | --- | --- |
| ENST00000425566.1 | 14.51 | 73.4 | RPL23AP87 | ribosomal protein L23a pseudogene 87 |
| ENST00000401722.7 | 15.16 | 77.1 | SLC25A3 | solute carrier family 25 (mitochondrial carrier; phosphate carrier), member 3 |
| ENST00000556083.1 | 17.07 | 76.6 | ACTN1 | actinin, alpha 1 |
| ENST00000409600.5 | 17.22 | 91 | BZW1 | basic leucine zipper and W2 domains 1 |
| ENST00000495596.5 | 18.08 | 73.4 | ATP5G2 | ATP synthase, H+ transporting, mitochondrial Fo complex subunit C2 (subunit 9) |
| ENST00000411857.2 | 18.21 | 219.1 | HNRNPA1P54 | heterogeneous nuclear ribonucleoprotein A1 pseudogene 54 |
| ENST00000500728.2 | 18.21 | 73 | LRPAP1 | LDL receptor related protein associated protein 1 |
| ENST00000359856.10 | 18.22 | 123.8 | ARPC5 | actin related protein 2/3 complex subunit 5 |
| ENST00000250559.13 | 18.25 | 111.3 | RAP1B | RAP1B, member of RAS oncogene family |
| ENST00000493763.5 | 18.37 | 76.1 | RPL7L1 | ribosomal protein L7 like 1 |
| ENST00000426371.2 | 18.49 | 99.4 | HNRNPA1P40 | heterogeneous nuclear ribonucleoprotein A1 pseudogene 40 |
| ENST00000343813.9 | 18.53 | 65.9 | ICMT | isoprenylcysteine carboxyl methyltransferase |
| ENST00000398752.10 | 18.82 | 106.4 | ATP5A1 | ATP synthase, H+ transporting, mitochondrial F1 complex, alpha subunit 1, cardiac muscle |
| ENST00000394670.8 | 18.84 | 91.3 | ZNF207 | zinc finger protein 207 |
| ENST00000308580.11 | 18.9 | 75.4 | TMOD3 | tropomodulin 3 |
| ENST00000335508.10 | 18.92 | 80.4 | SF3B1 | splicing factor 3b subunit 1 |
| ENST00000338970.10 | 18.96 | 109.1 | RPL14 | ribosomal protein L14 |
| ENST00000251527.9 | 19.1 | 72.9 | ESYT2 | extended synaptotagmin-like protein 2 |
| ENST00000337179.9 | 19.29 | 73.6 | NUDT4 | nudix hydrolase 4 |
| ENST00000361183.7 | 19.4 | 100.9 | CAPZA2 | capping protein (actin filament) muscle Z-line, alpha 2 |
| ENST00000369851.5 | 19.5 | 129.6 | GNAI3 | guanine nucleotide binding protein (G protein), alpha inhibiting activity polypeptide 3 |
| ENST00000258439.7 | 19.54 | 75.8 | TMEM127 | transmembrane protein 127 |
| ENST00000265044.6 | 19.54 | 87.6 | SSR3 | signal sequence receptor, gamma (translocon-associated protein gamma) |
| ENST00000359890.7 | 19.54 | 87.4 | RBM23 | RNA binding motif protein 23 |
| ENST00000334660.9 | 19.66 | 87.7 | CHP1 | calcineurin-like EF-hand protein 1 |
| ENST00000607016.1 | 19.92 | 86.8 | NUDT3 | nudix hydrolase 3 |
| ENST00000261318.4 | 19.99 | 91 | C12orf49 | chromosome 12 open reading frame 49 |
